# Genetic characterization of extended-spectrum β-Lactamase- and carbapenemase-producing *Escherichia coli* isolated from Egyptian hospitals and environments

**DOI:** 10.1371/journal.pone.0255219

**Published:** 2021-07-23

**Authors:** Soha El-Shaer, Shaymaa H. Abdel-Rhman, Rasha Barwa, Ramadan Hassan

**Affiliations:** Microbiology and Immunology Department, Faculty of Pharmacy, Mansoura University, Mansoura, Egypt; Imam Abdulrahman Bin Faisal University, SAUDI ARABIA

## Abstract

Over the past decades, *Escherichia coli* (*E*. *coli*) have acquired extensive resistance to antibiotics; especially β- lactams. This study aimed to investigate the frequency of Extended-spectrum β-lactamase (ESBL) and carbapenemase producers among *E*. *coli* isolates and their correlation with serotypes, phylogenetic background, and pathogenicity associated islands. A total of 105 *E*. *coli* strains were isolated and subjected to antimicrobial susceptibility testing against β-lactam antibiotics. All isolates showed a high resistance profile. Resistant isolates were tested for ESBL and carbapenemase production. Fifty-three and 18 isolates were positive for ESBL and carbapenemase producers, respectively. ESBL and carbapenemase genes were detected by PCR. *TEM* gene was the most prevalent gene among all isolates followed by *SHV* and *CTX-M15*. In carbapenemase-producers, *OXA-48* and *IMP* were the predominant genes. Enteropathogenic *E*. *coli* (EPEC) and Enterohemorrhagic *E*. *coli* (EHEC) were the major producers of ESBL and carbapenemase, respectively as indicated by serodiagnosis. They were further assessed for the presence of pathogenicity islands (PAIs) and phylogenetic background. The most predominant DEC PAI and ExPEC PAI were HPI and IICFT073. Most clinically ESBL-producers were group D and B2 while environmentally ones were group B1 and A. On contrary, clinically carbapenemase-producers belonged to group C and D. In conclusion, our study confirms the importance of phylogenetic group D, B2, and C origin for antibiotic resistance in *E*. *coli*. Ultimately, our findings support the fact that environmental isolates contribute to the local spread of *E*. *coli* pathogenicity in Egypt and these isolates maybe serve as reservoirs for transmission of resistance.

## Introduction

*Escherichia coli* is the most frequently isolated bacteria from microbiology laboratories [1]. Although *E*. *coli* is a member of normal gut microbiota, some isolates are pathogenic and may cause diarrhea and extra-intestinal disorders in humans [2].

*E*. *coli* can be classified into three subtypes from human health perspective [3]. First, commensal isolates colonizing the gut of healthy individuals. The second one is the diarrheagenic (DEC) isolates that cause diarrhea and differs according to strain virulence. Finally, extra-intestinal pathogenic *E*. *coli* (ExPEC) are similar to commensal ones in colonizing the human gut, but they can survive well in extra-intestinal environments causing serious human diseases.

The pathogenic behavior of a bacterial strain can be determined by assessing the virulence factor (VFs) collection and/or the phylogenetic background. *E*. *coli* is classified into eight phylogroups: A, B1, B2, C, D, E, F, and *Escherichia* cryptic clade I depending on the new quadruplex PCR-based method [4]. Genetically, ExPEC carries many VFs which allow them to avoid or subvert host defenses, colonize within anatomical sites, and/or induce host inflammatory response, thereby causing a disease status. These VFs form clusters named pathogenicity-associated island markers (PAIs) located in chromosome and/or plasmids [5]. The PAIs contribute to the transmission of many genes that help bacteria survive and cause diseases.

The development of antimicrobial resistance in *E*. *coli* is one of the biggest challenges in public health [6]. *E*. *coli* exhibits resistance to variable antibiotics, mainly extended-spectrum β-lactams (ESBLs) and carbapenem. ESBLs producers are isolates that are resistant to penicillins and cephalosporins [7]. They were observed either as variants of *TEM* and *SHV* or the CTX-M enzyme. It is more prevalent in environmental isolates suggesting that the environment is a reservoir of ESBL-producers [8]. This is a major contributor to increased antibiotic load, increased therapeutic cost, poor health outcomes, and limited therapeutic choices [9].

Carbapenemase producers are isolates with resistance to cephamycins and carbapenems via the expression of a variety of genes including *KPC*, *OXA-48*, *IMP*, *VIM*, and *NDM* [10]. The intestinal microbiota, feces, and rectal swabs are all typical reservoirs of carbapenemase production in hospital settings [11].

Resistance accumulation and dissemination in clinical and environmental *E*. *coli* underlined the necessity of developing suitable strategies to tackle antimicrobial resistance, where it is difficult to anticipate the development of novel antimicrobial agents. Therefore, this study aims to investigate the frequency of ESBL and/or carbapenemase producers in clinical and environmental *E*. *coli* isolates and to identify their correlation with phylogenetic groups, serotypes and PAIs.

## Material and methods

### Bacterial isolates

A total of 450 specimens were collected, 285 clinical specimens were obtained from different clinical sources from seven different hospitals in Mansoura, Egypt (Urology and Nephrology Center, Mansoura International Hospital, Mansoura Emergency Hospital, Mansoura University Hospital, Gastroenterology Center, Burns, and Cosmetics Center, Microbiology diagnostic Infection Control Unit). These specimens were 107 urinary isolates from UTIs (Urinary Tract Infections), 78 from rectal swabs from patients with intestinal disorder and 100 isolates from surgical wounds. A single isolate per patient is included in this study. In addition, 165 environmental specimens were obtained from a variety of sources (88 samples from different butchers’ shops and public supermarkets, 42 from feces of healthy humans, 15 from dairy products and 20 samples from different types of water and sludge) in Mansoura, Egypt. Each sample was transferred on ice to the microbiology laboratory. The bacteria isolates were identified biochemically as described by Mahon et al. [12]. The study was approved by The Research Ethics Committee, Faculty of Pharmacy, Mansoura University, Egypt (Code Number: 2015–58). The primary isolation for the clinical specimens was not performed specifically for the purposes of the study, the specimens were collected from the Infection control units at the hospitals where they took the patient consent approval for using these specimens in research work. All patient data were anonymous.

### Determination of antimicrobial sensitivity pattern of *E*. *coli* isolates

The antimicrobial sensitivity test of each isolate was carried out by the Kirby-Bauer disc diffusion technique according to CLSI [13] against β-lactam antibiotics (amoxicillin-clavulanic, ceftazidime, cefotaxime, ceftriaxone, cefepime, meropenem, and imipenem).

### Phenotypic detection of β-lactamases

#### Detection of ESBL enzymes

The double-disk synergy test (DDST) was used for phenotypic detection of ESBLs production in all *E*. *coli* isolates by following the CLSI guidelines [13] and as previously described [14]. Enhancement of the zone around any of the cephalosporin discs towards the disc containing clavulanic acid after 24 hrs incubation were recorded as positive for ESBLs production.

#### Detection of carbapenemase enzymes

Carbapenemase production was tested in all *E*. *coli* isolates by the Modified Hodge test according to the CLSI guidelines [13]. Plates with a clover leaf-type indentation at the intersection of the tested isolates and the sensitive *E*. *coli* isolate, within the zone of inhibition of the carbapenem susceptibility disk were considered positive plates, and hence, the tested isolates were carbapenemase producers.

### PCR for identification of β-lactamase genes

The boiling method was used for extracting genomic bacterial DNA [15]. Phenotypically detected ESBLs-producing *E*. *coli* isolates were tested by uniplex PCR using specific primers listed in Table 1 for *CTX-M15*, *SHV*, *TEM* [16], and multi-*TSO-O (OXA-1*, *-4*, *-30)* [17] genes.

**Table 1:** List of oligonucleotide primers used in this study.

Target genes	Type	Nucleotide sequence (5`to 3`)	Amplicon size (bp^c^)	Annealing Temp	References
**Set of primers used for amplification of ESBls encoding genes**					
***bla***_***CTX-M15***_	Fw^a^	GTGATACCACTTCACCTC	255	56	[16]
	Rv^b^	AGTAAGTGACCAGAATCAG			
***bla***_***SHV***_	Fw	ACTATCGCCAGCAGGATC	200	53	
	Rv	ATCGTCCACCATCCACTG			
***bla***_***TEM***_	Fw	GATCTCAACAGCGGTAAG	786	58	
	Rv	CAGTGAGGCACCTATCTC			
***bla***_***TSO-O (OXA-1*, *-4*, *-30)***_	Fw	GGCACCAGATTCAACTTTCAAG	564	60	[17]
	Rv	GACCCCAAGTTTCCTGTAAGTG			
**Set of primers used for amplification of carbapenemases encoding genes**					
***bla-***_***KPC***_	Fw	CATTCAAGGGCTTTCTTGCTGC	538	55	[17]
	Rv	ACGACGGCATAGTCATTTGC			
***bla-***_***IMP***_	Fw	TTGACACTCCATTTACDG	139		
	Rv	GATYGAGAATTAAGCCACYCT			
***bla-***_***VIM***_	Fw	GATGGTGTTTGGTCGCATA	390		
	Rv	CGAATGCGCAGCACCAG			
***bla***_***- OXA-48***_	Fw	GCTTGATCGCCCTCGATT	281	57	
	Rv	GATTTGCTCCGTGGCCGAAA			
***bla***_***- NDM-1***_	Fw	GGTTTGGCGATCTGGTTTTC	621	52	[18]
	Rv	CGGAATGGCTCATCACGATC			
**Set of primers used for amplification of ExPEC**^**d**^ **PAIs markers**					
**PAI I**_**536**_	Fw	TAATGCCGGAGATTCATTGTC	1800	55	[5]
	Rv	AGGATTTGTCTCAGGGCTTT			
**PAI II**_**536**_	Fw	CATGTCCAAAGCTCGAGCC	1000		
	Rv	CTACGTCAGGCTGGCTTTG			
**PAI III**_**536**_	Fw	CGGGCATGCATCAATTATCTTTG	200		
	Rv	TGTGTAGATGCAGTCACTCCG			
**PAI IV**_**536**_	Fw	AAGGATTCGCTGTTACCGGAC	300		
	Rv	TCGTCGGGCAGCGTTTCTTCT			
**PAI I**_**CFT073**_	Fw	GGACATCCTGTTACAGCGCGCA	930	56	
	Rv	TCGCCACCAATCACAGCGAAC			
**PAI II**_**CFT073**_	Fw	ATGGATGTTGTATCGCGC	400		
	Rv	ACGAGCATGTGGATCTGC			
**PAI I**_**J96**_	Fw	TCGTGCTCAGGTCCGGAATTT	400	53	
	Rv	TGGCATCCCACATTATCG			
**PAI II**_**J96**_	Fw	GGATCCATGAAAACATGGTTAATGGG	2300		
	Rv	GATATTTTTGTTGCCATTGGTTACC			
**Set of primers used for amplification DEC**^**e**^ **PAIs markers**					
**HPI**_***(irp2)***_	Fw	AAGGATTCGCTGTTACCGGAC	287	61	[19]
	Rv	TCGTCGGGCAGCGTTTCTTCT			
**O-islands *(efa/lifA)***	Fw	GAACAAAGAACATTTTCACCAGTTC	521	58	
	Rv	CTTTCAGGTGGGGAACCCG			
**She *(pic)***	Fw	ATTCTTCTGGCTGGCATTCC	606	57	
	Rv	CGGGATTAGAGACTATTGTTGC			
**EspC *(espC)***	Fw	GCTCAACTAAATATTGATAATGTATG	453	54	
	Rv	CCCAGCCCCAACCCTGAAAC			
**Tia *(tia)***	Fw	CCCTTCTGCATCCTTGTAAGACA	507	58	[20]
	Rv	TATAAGGGCGGTGATAAAAACG			
**Set of primers used for phylogenetic grouping (quadruplex PCR)**					
***chuA***	chuA.1b	ATGGTACCGGACGAACCAAC	288	59	[21]
	chuA.2b	TGCCGCCAGTACCAAAGACA			
***yjaA***	yjaA.1b	CAAACGTGAAGTGTCAGGAG	211		[4]
	yjaA.2b	AATGCGTTCCTCAACCTGTG			
***TspE4*.*C2***	TspE4C2.1b	CACTATTCGTAAGGTCATCC	152		
	TspE4C2.2b	AGTTTATCGCTGCGGGTCGC			
***arpA***	AceK.f	AACGCTATTCGCCAGCTTGC	400		[22]
	ArpA1.r	TCTCCCCATACCGTACGCTA			
**Set of primers used for phylogenetic grouping (duplex PCR)**					
**Group E**					
***arpA***	ArpAgpE.f	GATTCCATCTTGTCAAAATATGCC	301	57	[23]
	ArpAgpE.r	GAAAAGAAAAAGAATTCCCAAGAG			
***trpA***	trpBA.f	CGGCGATAAAGACATCTTCAC	489		[24]
	trpBA.r	GCAACGCGGCCTGGCGGAAG			
**Group C**					
***trpA***	trpAgpC.1	AGTTTTATGCCCAGTGCGAG	219	59	[23]
	trpAgpC.2	TCTGCGCCGGTCACGCCC			
***trpA***	trpBA.f	CGGCGATAAAGACATCTTCAC	489		[24]
	trpBA.r	GCAACGCGGCCTGGCGGAAG			

a**: Fw:** forward

b: **Rv:** reverse

c: **bp:** base pair

d: **ExPEC:** extra-intestinal pathogenic *E*. *coli*

e: **DEC:** Diarrheagenic *E*. *coli*.

*E*. *coli* isolates that were phenotypically characterized as resistant to carbapenems (imipenem and meropenem) were analyzed by PCR for different carbapenemase encoding genes (*IMP*, *VIM*, *KPC*, *NDM*-1, and *OXA-48*) [17, 18] using primers listed in Table 1. *NDM* and OXA were detected by uniplex PCR. Other carbapenemase genes were detected by multiplex PCR The temperature profile and the PCR conditions for PAIs primers were conducted as described in (S1 Table in S1 File) [16–18].

### *E*. *coli* serotyping

The isolates were serologically identified according to [25] by using rapid diagnostic *E*. *coli* antisera sets (DENKA SEIKEN Co., Japan) for diagnosis of the Enteropathogenic types.

### Molecular characterization of PAI and determination of phylogenetic groups

The isolates were assessed for the presence of pathogenicity islands (PAIs). Eight PAIs including PAI I536, PAI II536, PAI III536, PAI IV536, PAI ICFT073, PAI IICFT073, PAI IJ96, and PAI IIJ96 belonging to ExPEC were detected by duplex PCR [5]. Besides, Five PAIs portable genes including HPI (*irp2*), Tia (*tia*), O-island (*efa/lifA*), She (*pic*), and EspC (*espC*) PAIs belonging to Diarrheagenic *E*. *coli* (DEC) were detected by uniplex PCR using primers in Table 1 [19, 20]. The temperature profile and the PCR conditions for PAIs primers were conducted as described in (S1 Table in S1 File). Besides, these isolates were classified phylogenetically into eight groups: A, B1, B2, D, F, clade I (by quadruplex PCR) [4, 21, 22], C, and E (by duplex PCR assay) [23, 24] using primers in Table 1. The cycling conditions incorporated; initial denaturing at 95°C for 5 mins, then 40 cycles of denaturation at 95°C for 30 sec, annealing for 30 sec at (57°C for group E, 59°C for quadruplex and group C assays) and extension at 72°C for 1 min, then the program ended with final extension cycle at 72°C for 5 mins.

### Statistical analysis

Graph Pad Prism software package (version 5.01) was used for statistical analysis of the data correlations applying Fisher’s exact test and Chi-square test. The level of significance was set at a p-value < 0.05.

## Results

### Determination of antimicrobial sensitivity pattern

A total of 105 *E*. *coli* isolates were identified (72 from clinical sources and 33 from environmental sources) (**S2** and **S3 Tables in S1 File**). All isolates showed extreme resistance to the tested antimicrobial agents except nearly carbapenems (**S4 Table in S1 File**). For the clinical isolates, resistance against cefotaxime (84.7%, 61 isolates) and ceftriaxone (75%, 54 isolates) were the most prevalent. In contrast, only 12.5% (nine isolates) and 6.9% (five isolates) of the isolates were resistant to meropenem and imipenem, respectively. Totally, 75% (54 isolates) of isolates exhibited resistance to at least three antibiotics (P< 0.0001).

**Fig 1:**
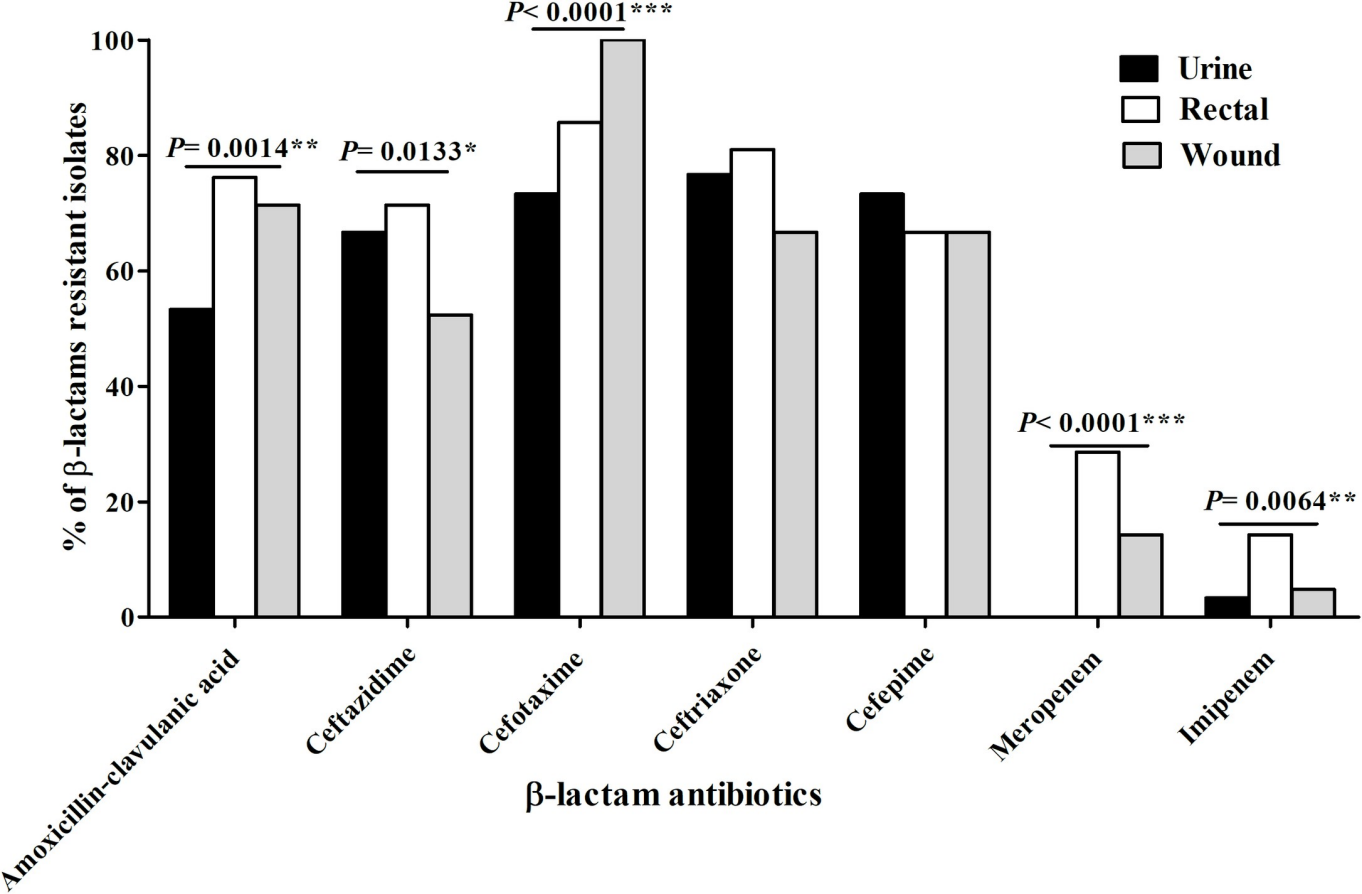
Comparison of β-lactams resistance level among the clinical and environmental *E*. *coli* isolates. (*significant, P< 0.05 and ***highly significant, P< 0.0001).

Regarding environmental isolates, resistance against cefepime (93.9%,31 isolates) and cefotaxime (81.8%, 27 isolates) were predominant as illustrated in **Fig 1**. All environmental isolates were sensitive to imipenem. Generally, 82% of isolates (27 isolates) exhibited resistance to at least three antibiotics (P< 0.0001).

### Qualitative detection of ESBLs

**Fig 2:**
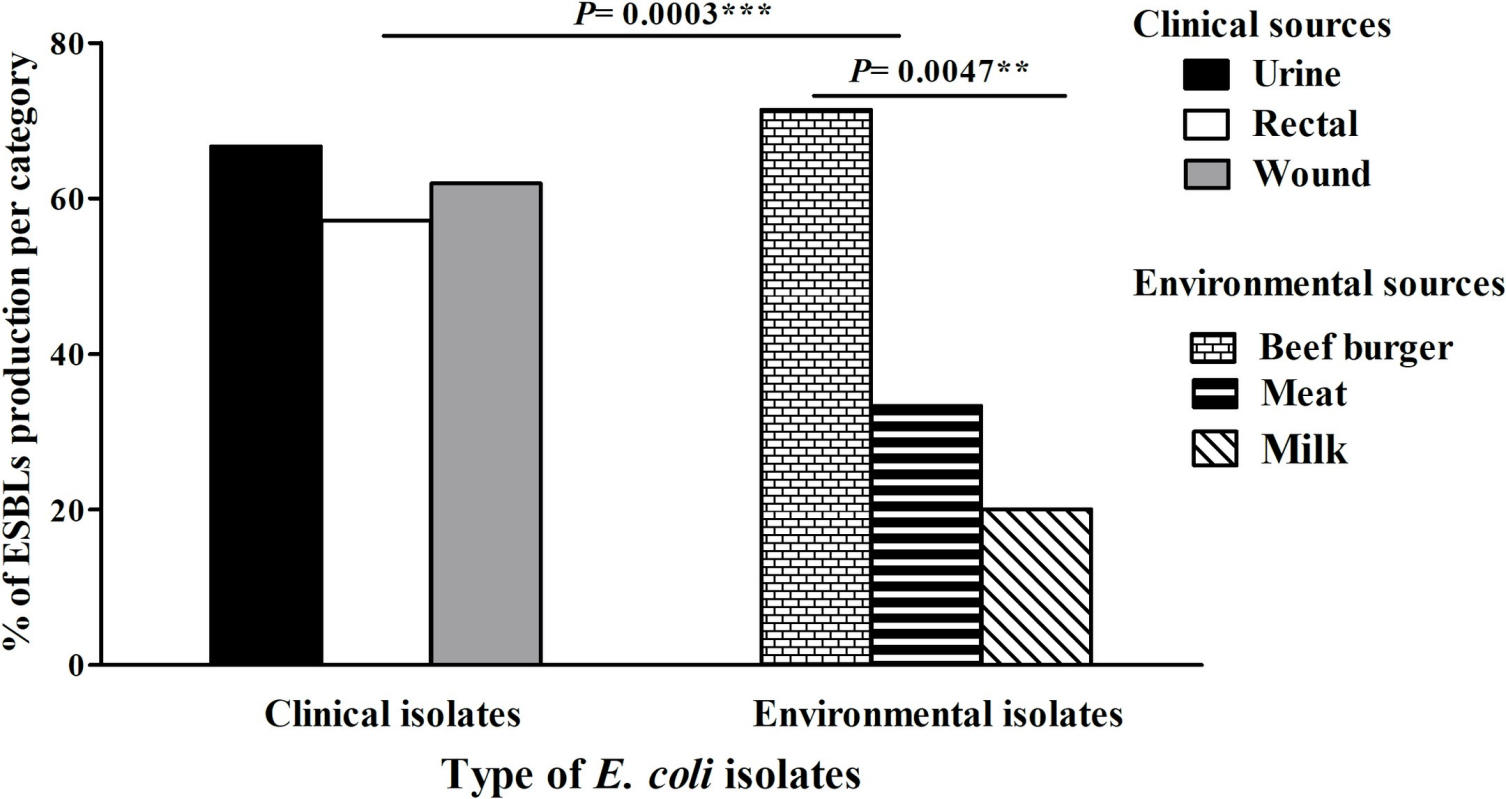
Distribution of extended-spectrum β-lactamase (ESBLs) enzymes among clinical and environmental *E*. *coli* isolates. (**: moderately significant, P< 0.01, ***: highly significant, P< 0.0001).

Fifty-three isolates (50.5%) were classified as ESBL-producers including 45 (62.5%) clinical and eight (24.2%) environmental isolates (P = 0.003). The identified ESBL-producers were distributed among the studied clinical sources with the majority originated from UTIs. In contrast, ESBLs producing environmental isolates were restricted to three sources including beef burger (71.4%), meat (33.3%), and milk (20%) with P value = 0.0047 as illustrated in **Fig 2.**

### Modified Hodges test

Modified Hodges test was performed on 18 carbapenem-resistant *E*. *coli*. All these isolates showed positive modified Hodges test as compared to the negative control.

### PCR identification of β-lactamase encoding genes

**Fig 3:**
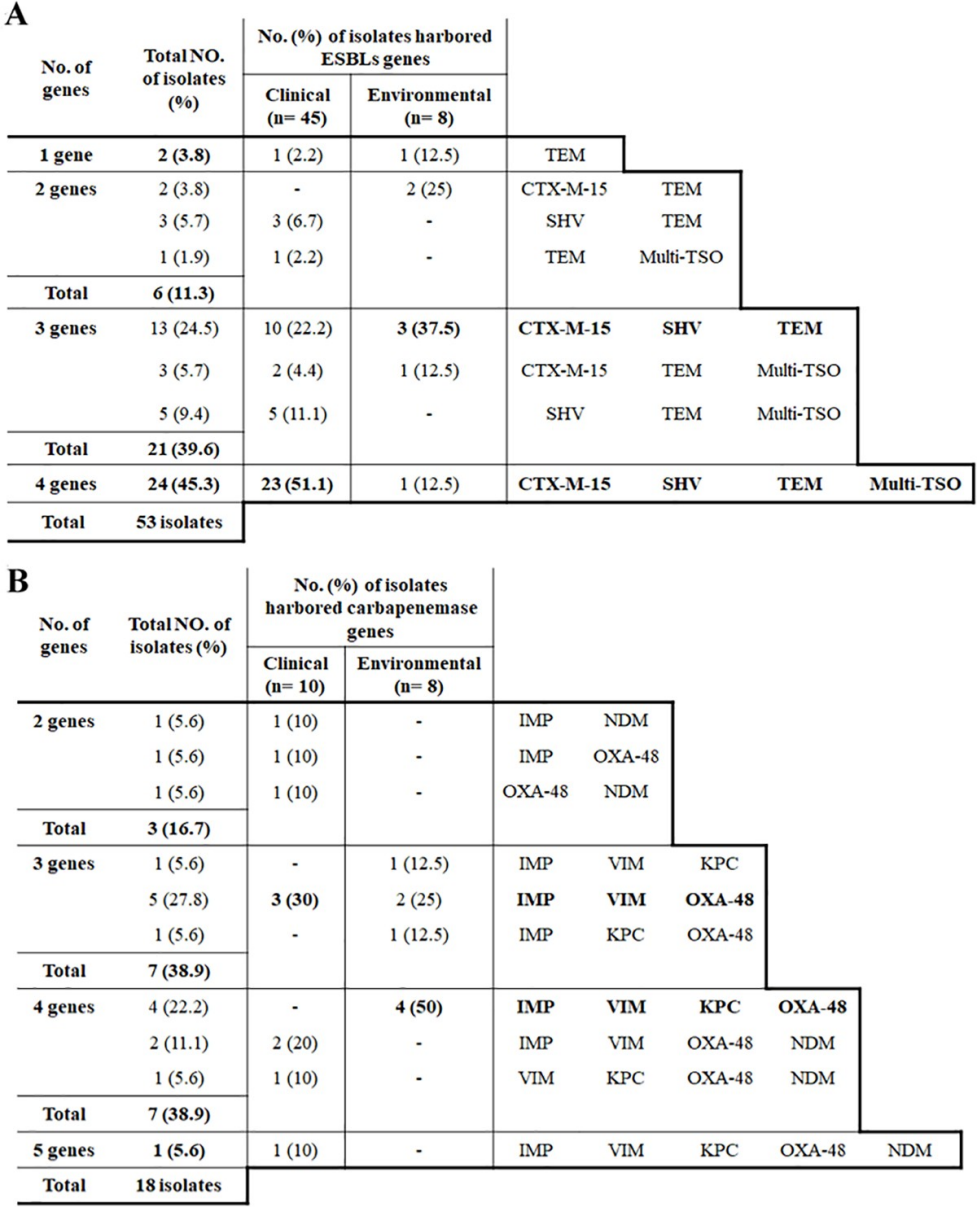
Hierarchical diagram of β-lactamase *bla* genes. A): Hierarchical diagram of ESBL encoding genes among 45 clinical and 8 environmental isolates based on possession of single or multiple *bla* gene combinations. B): Hierarchical diagram of carbapenemase encoding genes among 10 clinical and 8 environmental isolates based on possession of multiple *bla* gene combinations.

PCR analysis of four ESBL genes **(S5 Table in S1 File)** revealed that TEM was the most predominant (100%), while multi-*TSO-O* was the least detected one (62.3%). The distribution of ESBL genes among clinical and environmental isolates revealed that only *SHV* and multi-*TSO-O* showed a significant difference (P<0.0001). Various ESBL gene combinations were predominant where 96.2% of the isolates harbored ≥2 genes. The combination of the four tested ESBL genes were common among clinical isolates (51.1%) while (*CTX-M15*+*SHV*+*TEM*) was predominant among environmental isolates (3/8 isolates) **(Fig 3A).**

Regarding carbapenemases genes **(S6 Table in S1 File)**, *IMP* and *OXA-48* were the most prevalent genes (89%), while *NDM* was the least detected one (33.3%). *NDM* was detected only in the clinical isolates. Statistical analysis of carbapenemase encoding genes among clinical and environmental isolates showed significance (P<0.001) for all genes except *OXA-48*. Ten carbapenemase encoding gene combinations were detected. Six of them belonged to the clinical isolates, while three unique combinations were found in the environmental isolates **(Fig 3B).**

Five isolates (three clinical and two environmental isolates) were classified as both ESBL and carbapenemase co-producers. All ESBL and carbapenemase co-producers harbored *TEM*, CTX-M15, and *IMP*. *SHV* was detected in 80% of the isolates, 60% carried multi-*TSO-O*, *OXA-48*, and *VIM*, while *KPC* and *NDM* were carried by two isolates each **(S5 and S6 Tables in S1 File**)

### Serodiagnosis

All ESBL- and carbapenemase-producers were serologically identified. In ESBL-producers, EPEC was the predominant pathotype in 25 isolates, while (enteroinvasive *E*. *coli*) EIEC was the least detected only found in two clinical isolates **(S5 and S6 Tables in S1 File**).

Regarding carbapenemase-producers, EHEC represents the most pervasive pathotype (50% of isolates), followed by EPEC (27.8%), (enterotoxigenic *E*. *coli*) ETEC, and EIEC each represented by two isolates. EIEC was restricted to clinical isolates only, one of them was ESBLs and carbapenemases coproducer.

Concerning serotypes, 24 serotypes were detected within pathotypes. Eight serotypes were shared between the clinical and environmental isolates, 13 serotypes belonged to clinical isolates and only two serotypes were unique in environmental ones. The clinical and environmental ESBL-producers comprised 14 and two serotypes, respectively, in addition to six shared serotypes. The prevalent serotype was O15:H2 (9.4%) followed by O2:H6, O26:H11, O2:H6, O127:H6, and O55:H7 (7.5% each). Carbapenemase-producers shared 3 serotypes, while five serotypes were unique to individual isolates. There were two serotypes restricted to carbapenemase-producers: O103:H2 (16.7%) and O121:H7 (5.6%).

Eleven, twelve, four, and one serotypes were found in EPEC, EHEC, ETEC, and EIEC, respectively. Besides, the prevalent serotypes included O15:H2, O91:H21, O127:H6 and O124 in EPEC (18.5%), EHEC (18.5%), ETEC (55.5%) and EIEC (100%), respectively. The majority of clinically originated ESBL-producers belonged to EPEC (P<0.0001) and EHEC (P≤0.001). For the clinical carbapenemase-producers, *KPC* was associated with EHEC (P<0.0001) while *NDM* was present in all pathotypes significantly except ETEC. However, in the environmental ones, *VIM* was significantly associated with EHEC and EPEC, while *KPC* and *OXA-48* were distributed in all pathotypes.

### Molecular detection of pathogenicity island markers (PAIs)

Table 2 illustrates the distribution of DEC and ExPEC PAI markers among *E*. *coli* isolates. 94.5% of the clinical isolates and all the environmental isolates, in both ESBL and carbapenemase-producers, carried PAI markers. The major DEC PAI marker was HPI (*irp2*) (90.9%). In contrast, EspC (*espC*) was the least detectable PAI marker, it was found only in carbapenemase-producers (11.1%).

**Table 2:** Distribution of clinical and environmental *E*. *coli* isolates with DEC and ExPEC PAI markers.

PAI		No. (%) of ESBL-producers		No. (%) of Carbapenemase producers	
		Clinical (n = 45)	Environmental (n = 8)	Clinical (n = 10)	Environmental (n = 8)
**DEC**	**HPI (*irp2*)**	43 (95.5)***	3 (37.5)	10 (100)***	4 (50)
	**Tia (*tia*)**	14 (31.1)	5 (62.5)***	3 (30)	2 (25)
	**O-island (*efa/lifA*)**	4 (8.8)**	0 (0)	3 (30)	0 (0)
	**SHE (*pic*)**	4 (8.8)*	1 (12.5)	1 (10)	2 (25)
	**EspC (*espC*)**	0 (0)	0 (0)	1 (10)	1 (12.5)
**EX-PEC**	**PAI I**_**536**_	2 (4.4)*	0 (0)	0 (0)	0 (0)
	**PAI II**_**536**_	2 (4.4)	1 (12.5)	1 (10)	1 (12.5)
	**PAI III**_**536**_	23 (51.1)***	7 (87.5)	9 (90)**	5 (62.5)
	**PAI IV**_**536**_	38 (84.4)***	6 (75)	10 (100)*	7 (87.5)
	**PAI I**_**CFT073**_	24 (53.3)***	4 (50)	7 (70)***	2 (25)
	**PAI II**_**CFT073**_	40 (88.9)***	7 (87.5)	10 (100)	8 (100)
	**PAI I**_**J96**_	18 (40.0)***	2 (25)	3 (30)*	1 (12.5)
	**PAI II**_**J96**_	0 (0)	0 (0)	0 (0)	0 (0)

*: significant, *P*< 0.05

**: moderately significant, *P*< 0.01 and

***: highly significant, *P*< 0.0001.

Regarding ExPEC PAIs, the most prevalent marker among β-lactamase producers was PAI II_CFT073_ (98.5%) followed by PAI IV_536_ (92.4%). PAI II_J96_ was absent from all isolates.

**Fig 4:**
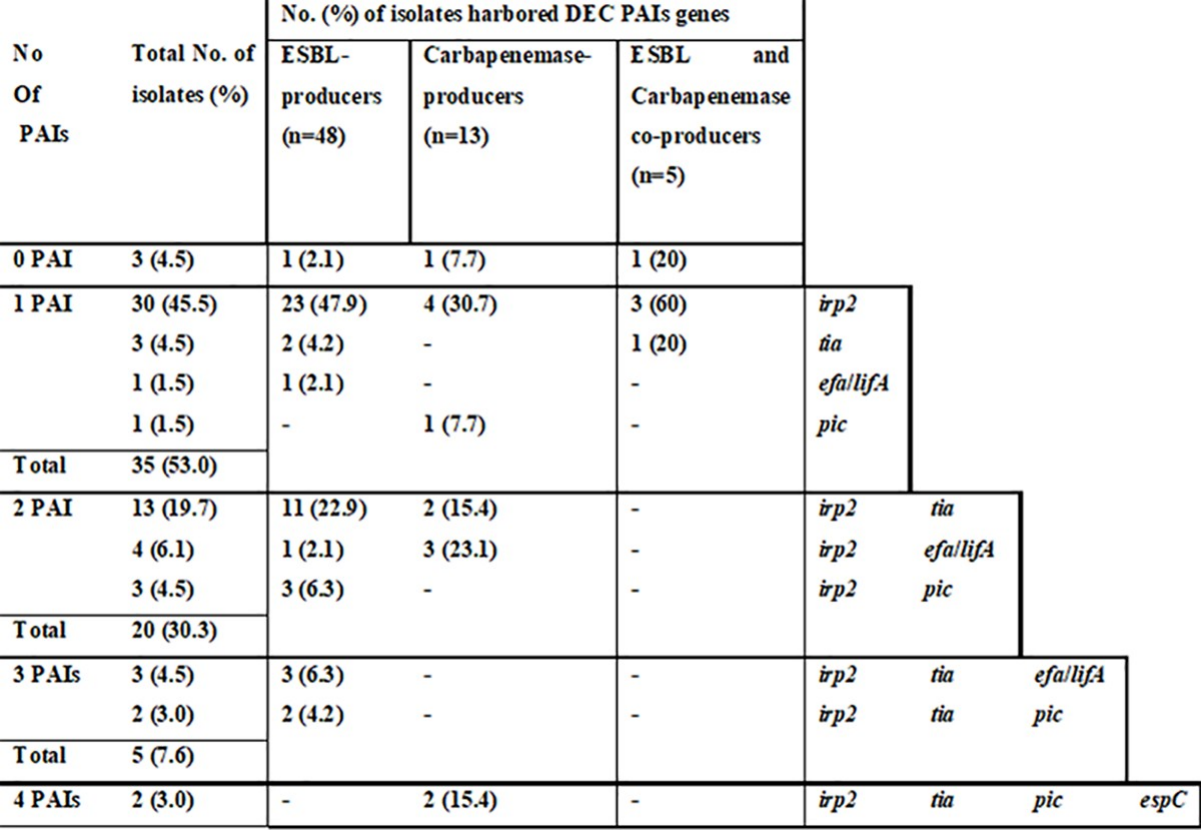
Hierarchical diagram of diarrheagenic *Escherichia coli* pathogenicity island markers among ESBL and carbapenemase-producers based on non-possession or possession of single/multiple combinations of pathogenicity island markers. PAIs: pathogenicity islands.

**Fig 5:**
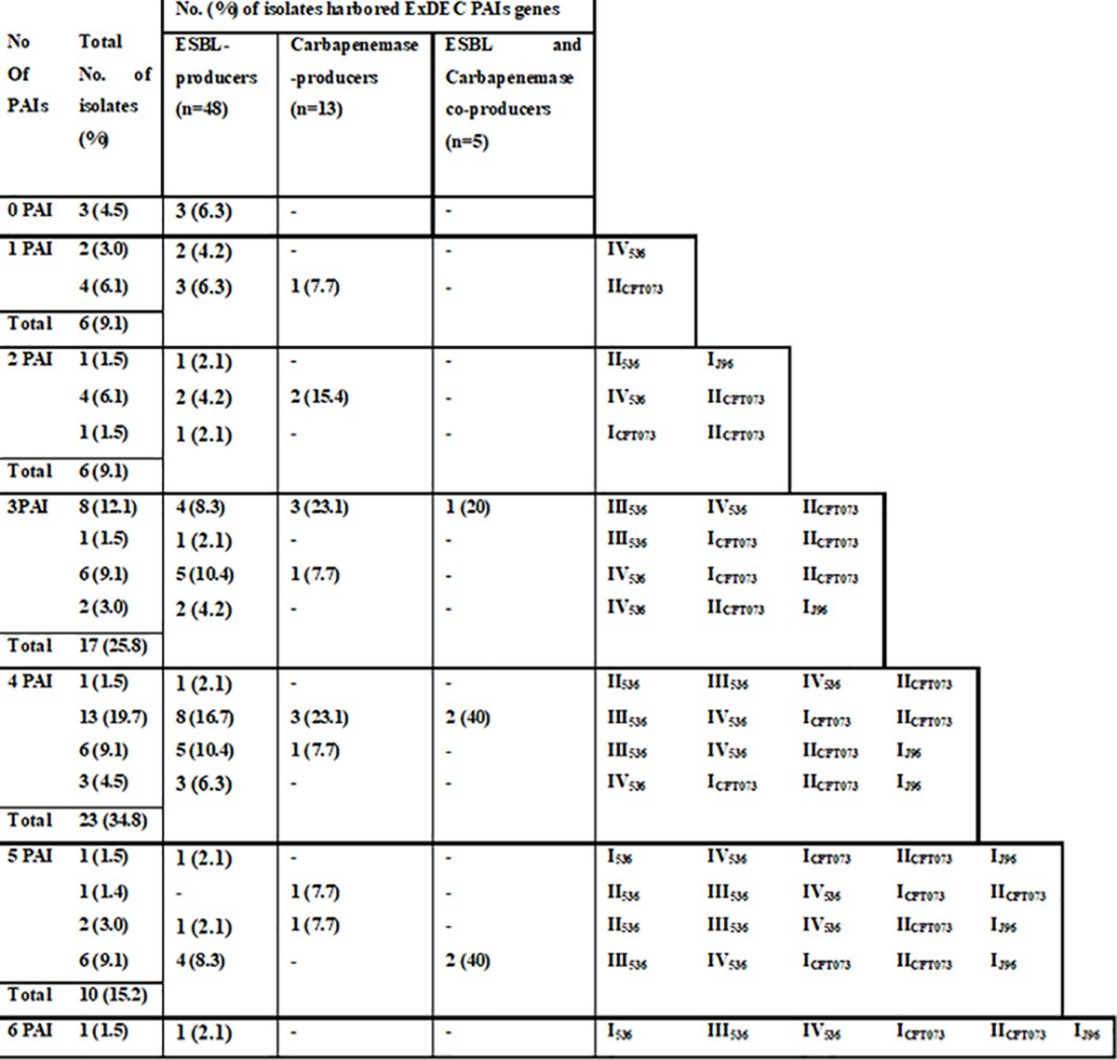
Hierarchical diagram of extra-intestinal *Escherichia coli* pathogenicity island markers among ESBL and carbapenemase-producers based on non-possession or possession of single/multiple combinations of pathogenicity island markers. PAIs: pathogenicity islands.

Different PAIs combinations were detected among the tested *E*. *coli* isolates. A single DEC PAI marker gene was detected in 35 isolates **(Fig 4)** while, 57 isolates harbored ≥2 ExPEC PAIs **(Fig 5)**. Six patterns designed (*irp2*+*tia)* were prevalent in ESBL and carbapenemase-producers. Three unique PAI combinations belonged to ESBL-producers (16.8%). Sixteen ExPEC PAIs marker combinations were detected. The major combination in ESBL (16.7%) and carbapenemase-producers (23.1%) was (PAI III_536_+PAI IV_536_+PAI I_CFT073_+PAI II_CFT073_). Nine unique combinations were found in ESBL-producers. In contrast, only one unique ExPEC combination was detected in carbapenemase-producers. Moreover, 61 isolates carried both DEC and ExPEC PAIs forming 37 combination patterns **(**Table 3**)**. The most frequent combination (8.3%) was (*irp2*, PAI III_536_, PAI IV_536_, PAI I_CFT037_, and PAI II_CFT037_).

**Table 3:** Distribution of diarrheagenic and extra-intestinal pathogenicity island marker combinations among ESBLs and carbapenemase-producing *E*. *coli* isolates.

No.^a^ of PAIs	DEC^b^ and ExPEC^c^ PAI^d^ marker combinations	No. (%) of isolates harbored both PAI markers			Total No. of isolates (%) (n = 61)
		ESBL-producers (n = 47)	Carbapenemase-producers (n = 10)	ESBL and Carbapenemase co-producers (n = 4)	
**2**	*irp2*, PAI IV_536_	1 (2.1)	-	-	1 (1.6)
	*irp2*, PAI II_CFT037_	2 (4.2)	1 (10)	-	3 (4.9)
**3**	*irp2*, PAI II_536_, PAI I_J96_	1 (2.1)	-	-	1 (1.6)
	*irp2*, PAI IV_536_, PAI II_CFT037_	2 (4.2)	1 (10)	-	3 (4.9)
	*irp2*, PAI I_CFT037_, PAI II _CFT037_	1 (2.1)	-	-	1 (1.6)
	*irp2*, *efa/lifA*, PAI IV536	1 (2.1)	-	-	1 (1.6)
**4**	***irp2*,** PAI III_536_, PAI IV_536_, PAI II_CFT037_	3 (6.3)	**-**	-	3 (4.9)
	*irp2*, PAI IV_536_, PAI I_CFT037_, PAI II_CFT037_	2 (4.2)	-	-	2 (3.2)
	*irp2*, *tia*, *efa/lifA*, PAI II_CFT037_	1 (2.1)	-	-	1 (1.6)
	*tia*, PAI III_536_, PAI IV_536_, PAI II_CFT037_	2 (4.2)	1 (10)	-	3 (4.9)
	*Pic*, PAI III_536_, PAI IV_536_, PAI II_CFT037_	-	-	1 (25)	1 (1.6)
**5**	*irp2*, PAI III_536_, PAI IV_536_, PAI I_CFT037_, PAI II_CFT037_	3 (6.3)	1 (10)	1 (25)	5 (8.2)
	*irp2*, PAI III_536_, PAI IV_536_, PAI II_CFT037_, PAI I_J96_	2 (4.2)	-	-	2 (3.2)
	*irp2*, PAI IV_536_, PAI I_CFT037_, PAI II_CFT037_, PAI I_J96_	2 (4.2)	-	-	2 (3.2)
	*irp2*, *tia*, PAI III_536_, PAI IV_536_, PAI II_CFT037_	1 (2.1)	-	-	1 (1.6)
	*irp2*, *tia*, PAI IV_536_, PAI I_CFT037_, PAI II_CFT037_	2 (4.2)	1 (10)	-	3 (4.9)
	*irp2*, *tia*, PAI IV_536_, PAI II_CFT037_, PAI I_J96_	2 (4.2)	-	-	2 (3.2)
	*irp2*, *efa/lifA*, PAI III_536_, PAI IV_536_, PAI II_CFT037_	1 (2.1)	-	-	1 (1.6)
	*tia*, PAI III_536_, PAI IV_536_, PAI I_CFT037_, PAI II_CFT037_	1 (2.1)	-	-	1 (1.6)
**6**	*irp2*, PAI I_536_, PAI IV_536_, PAI I_CFT037_, PAI II_CFT037_, PAI I_J96_	1 (2.1)	-	-	1 (1.6)
	*irp2*, PAI II_536_, PAI III_536_, PAI IV_536_, PAI II_CFT037_, PAI I_J96_	1 (2.1)	1 (10)	-	2 (3.2)
	*irp2*, PAI III_536_, PAI IV_536_, PAI I_CFT037_, PAI II_CFT037_, PAI I_J96_	2 (4.2)	-	2 (50)	4 (6.5)
	*irp2*, *tia*, PAI III_536_, PAI IV_536_, PAI I_CFT037_, PAI II_CFT037_	2 (4.2)	-	-	2 (3.2)
	*irp2*, *tia*, PAI III_536_, PAI IV_536_, PAI II_CFT037_, PAI I_J96_	2 (4.2)	-	-	2 (3.2)
	*irp2*, *tia*, PAI IV_536_, PAI I_CFT037_, PAI II_CFT037_, PAI I_J96_	1 (2.1)	-	-	1 (1.6)
	*irp2*, *efa/lifA*, PAI III_536_, PAI IV_536_, PAI II_CFT037_, PAI I_J96_	-	1 (10)	-	1 (1.6)
	*irp2*, *pic*, PAI III_536_, PAI IV_536_, PAI I_CFT037_, PAI II_CFT037_	1 (2.1)	-	-	1 (1.6)
	*irp2*, *tia*, *efa/lifA*, PAI IV_536_, PAI I_CFT037_, PAI II_CFT037_	1 (2.1)	-	-	1 (1.6)
	*irp2*, *tia*, *pic*, PAI III_536_, PAI I_CFT037_, PAI II_CFT037_	1 (2.1)	-	-	1 (1.6)
	*irp2*, *tia*, *pic*, *espC*, PAI IV_536_, PAI II_CFT037_	-	1 (10)	-	1 (1.6)
**7**	*irp2*, *tia*, PAI III_536_, PAI IV_536_, PAI I_CFT037_, PAI II_CFT037_, PAI I_J96_	1 (2.1)	-	-	1 (1.6)
	*irp2*, *tia*, *efa/lifA*, PAI II_536_, PAI III_536_, PAI IV_536_, PAI II_CFT037_	1 (2.1)	-	-	1 (1.6)
	*irp2*, *tia*, *pic*, PAI III_536_, PAI IV_536_, PAI II_CFT037_, PAI I_J96_	1 (2.1)	-	-	1 (1.6)
	*irp2*, *efa/lifA*, PAI II_536_, PAI III_536_, PAI IV_536_, PAI I_CFT037_, PAI II_CFT037_	-	1 (10)	-	1 (1.6)
	*irp2*, *pic*, PAI III_536_, PAI IV_536_, PAI I_CFT037_, PAI II_CFT037_, PAI I_J96_	1 (2.1)	-	-	1 (1.6)
**8**	*irp2*, *tia*, *espC*, *pic*, PAI III_536_, PAI IV_536_, PAI I_CFT037_, PAI II_CFT037_	-	1 (10)	-	1 (1.6)
	*irp2*, *pic*, PAI I_536_, PAI III_536_, PAI IV_536_, PAI I_CFT037_, PAI II_CFT037_, PAI I_J96_	1 (2.1)	-	-	1 (1.6)

**a: NO.:** number

b: **DEC:** diarrheagenic *E*. *coli*

c: **ExPEC:** extra-intestinal pathogenic *E*. *coli*

d: **PAI:** pathogenicity island.

### Phylogenetic analysis

#### Phylogenetic analysis with relation to ESBL and carbapenemase-producers

Group D and B2 were found in 26 clinically originated ESBL-producers, while the remaining isolates were distributed among the groups. The environmental ESBL-producers belonged to groups A (25%), B1 (62.5%), and B2 (12.5%).

Five clinical carbapenemase-producers were group C, three were group D, and one isolate for each of groups E and U. The environmental carbapenemase-producers were distributed between four groups: B1 (37.5%), D (25%), A (25%), and B2 (12.5%).

**Fig 6:**
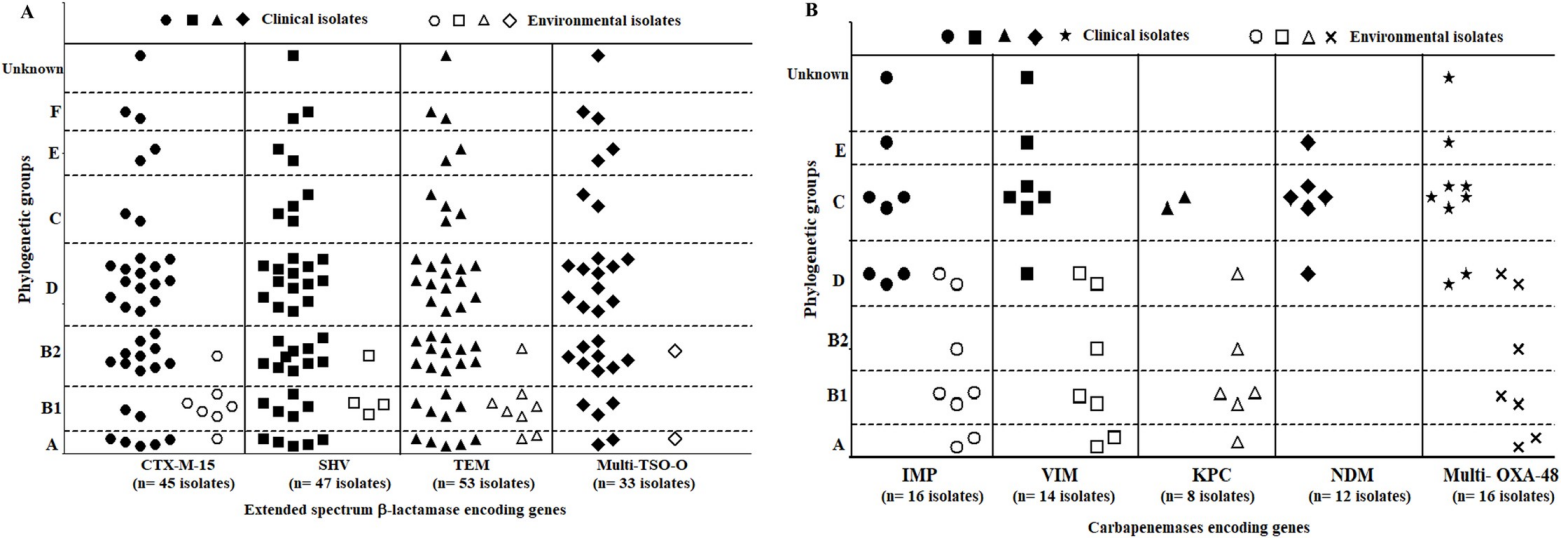
Distribution of β-lactamase *bla* genes among different phylogenetic groups. A) ESBL encoding genes among 53 *E*. *coli* isolates. B) Carbapenemase encoding genes among 18 *E*. *coli* isolates. Solid shapes: clinical isolates and empty shapes: environmental isolates.

Clinical isolates carrying ESBL genes were distributed within all phylogroups while the environmental isolates were distributed in A, B1, and B2 groups. ESBL tested genes were associated with Group D. Groups D and B2 were mainly represented by the clinical isolates, while the environmental isolates were common in group B1 as 62.5% of those harbored CTX-M15 and *TEM*, 37.5% for *SHV*, while multi-*TSO-O* isolates were equally distributed between groups A and B2 (12.5% each). All new phylogroups were restricted to clinical ESBL producers **(Fig 6A)**.

The carbapenemase-producers were distributed within all phylogroups except group F. It was found that groups A, B1 and B2 were only represented by environmental isolates, while groups C, E, and unknowns were solely clinical isolates **(Fig 6B)**. Group C comprised isolates mainly harboring *VIM* (28.6%), *NDM* (33.3%), and *OXA-48* (31.3%), while most of the isolates with *IMP* and *KPC* belonged to groups D (31.3%) and B1 (37.5%), respectively.

**Fig 7:**
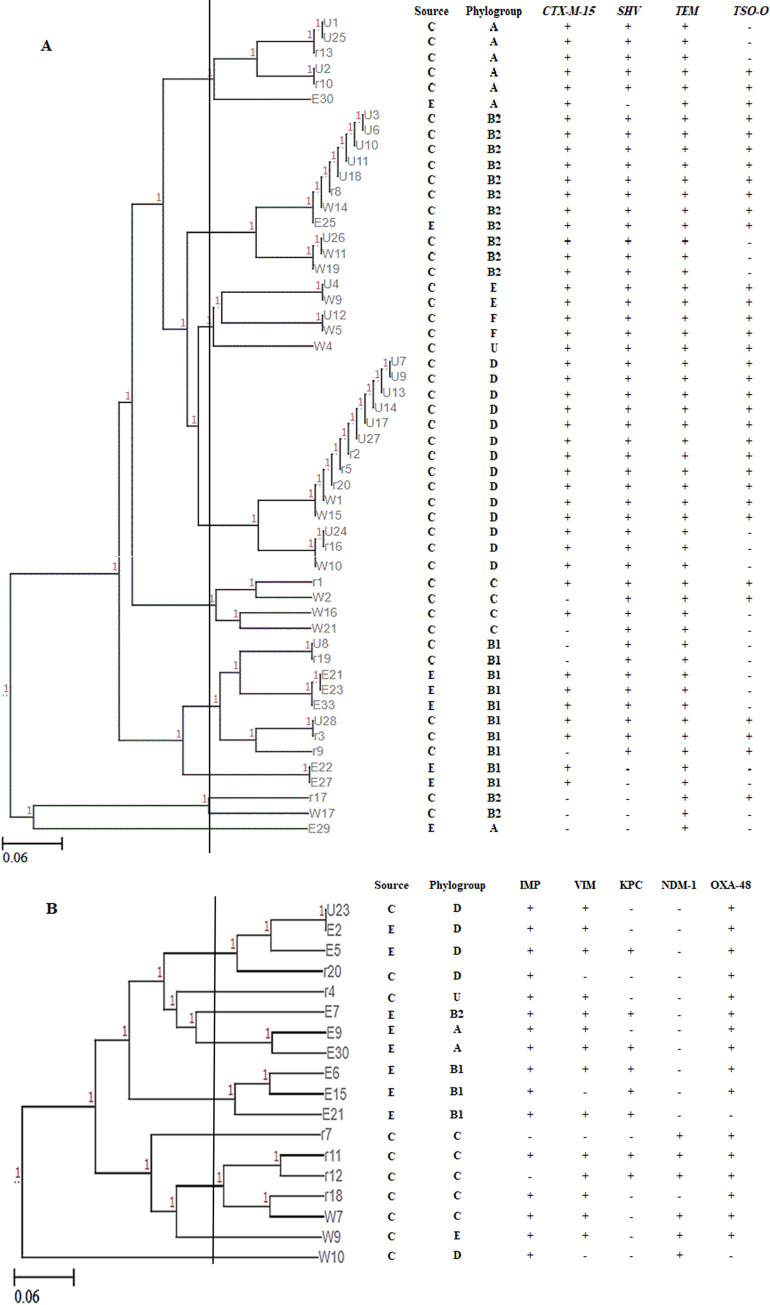
Dendrogram representing the relation between β-lactamase *bla* genes with different phylogroups. A) ESBL encoding genes among 53 *E*. *coli* isolates. B) Carbapenemase encoding genes among 18 *E*. *coli* isolates.

The relationship of the detected β-lactamase *bla* genes with phylogenetic groups was analyzed using UGMA program (Fig 7). The dendrogram showed that there was a high similarity of the genetic profile in the ESBL producers (Fig 7A) and carbapenemase producers (Fig 7B) of the same phylogroup at 65% cutoff. Moreover, all ESBL genes were found in isolates belonged to all phylogroups with the majority in B2 and D. Only one of the carbapenemase producers carried all the carbapenemases genes belonged to group C.

#### Phylogenetic relationship with PAI markers

The distribution of PAIs among β-lactams producers according to phylogenetic groups **(Table 4)** showed that group B2 was the predominant group among ESBL-producers, group C was prevalent in carbapenemase-producers while group D was pervasive in co-producers. HPI was predominant among groups B2 and D in ESBL-producers, group C in carbapenemase-producers, and group D in co-producers. The least prevalent DEC PAI was EspC where it was harbored by 2 isolates in group D and Clermont unknowns.

**Table 4:** Distribution of pathogenicity islands among clinical and environmental *E*. *coli* isolates, classified according to phylogenetic groups.

Phylogenetic group		N_T_ ^a^	N ^b^	Pathogenicity islands												
				ExPEC^c^ PAIs ^e^								DEC ^d^ PAIs ^e^				
				PAI I_536_	PAI II_536_	PAI III_536_	PAI IV_536_	PAI I_CFT073_	PAI II_CFT073_	PAI I_J96_	PAI II_J96_	HPI	Tia	O-island	She	EspC
Group A	• **ESBL**	**6**	**20**	**0**	**1**	**3**	**3**	**2**	**4**	**0**	**0**	**3**	**2**	**2**	**0**	**0**
	• **Carbapenemase**	**1**	**3**	**0**	**0**	**0**	**1**	**0**	**1**	**0**	**0**	**1**	**0**	**0**	**0**	**0**
	• **Co-producers**	**1**	**4**	**0**	**0**	**1**	**1**	**0**	**1**	**0**	**0**	**0**	**1**	**0**	**0**	**0**
Group B1	• **ESBL**	**9**	**40**	**0**	**1**	**6**	**5**	**4**	**8**	**3**	**0**	**7**	**3**	**0**	**3**	**0**
	• **Carbapenemase**	**2**	**3**	**0**	**0**	**0**	**0**	**0**	**1**	**0**	**0**	**1**	**0**	**0**	**1**	**0**
	• **Co-producers**	**1**	**4**	**0**	**0**	**1**	**1**	**1**	**1**	**0**	**0**	**0**	**0**	**0**	**0**	**0**
Group B2	• **ESBL**	**1**	**68**	**1**	**1**	**6**	**13**	**8**	**13**	**7**	**0**	**13**	**5**	**0**	**1**	**0**
	• **Carbapenemase**	**3**	**6**	**0**	**1**	**1**	**1**	**0**	**1**	**1**	**0**	**1**	**0**	**0**	**0**	**0**
	• **Co-producers**	**2**	**0**	**0**	**0**	**0**	**0**	**0**	**0**	**0**	**0**	**0**	**0**	**0**	**0**	**0**
Group C	• **ESBL**	**4**	**13**	**0**	**0**	**2**	**2**	**1**	**2**	**0**	**0**	**4**	**1**	**1**	**0**	**0**
	• **Carbapenemase**	**5**	**28**	**0**	**1**	**5**	**5**	**2**	**5**	**1**	**0**	**5**	**1**	**3**	**0**	**0**
	• **Co-producers**	**0**	**0**	**0**	**0**	**0**	**0**	**0**	**0**	**0**	**0**	**0**	**0**	**0**	**0**	**0**
Group D	• **ESBL**	**1**	**57**	**0**	**0**	**5**	**12**	**6**	**10**	**6**	**0**	**12**	**4**	**1**	**1**	**0**
	• **Carbapenemase**	**2**	**15**	**0**	**0**	**1**	**3**	**2**	**3**	**0**	**0**	**2**	**2**	**0**	**1**	**1**
	• **Co-producers**	**3**	**12**	**0**	**0**	**2**	**2**	**2**	**2**	**2**	**0**	**2**	**0**	**0**	**0**	**0**
Group E	• **ESBL**	**1**	**9**	**0**	**0**	**1**	**2**	**2**	**2**	**0**	**0**	**1**	**1**	**0**	**0**	**0**
	• **Carbapenemase**	**0**	**4**	**0**	**0**	**1**	**1**	**1**	**1**	**0**	**0**	**0**	**0**	**0**	**0**	**0**
	• **Co-producers**	**1**	**5**	**0**	**0**	**1**	**1**	**1**	**1**	**0**	**0**	**1**	**0**	**0**	**0**	**0**
Group F	• **ESBL**	**2**	**8**	**0**	**0**	**1**	**1**	**1**	**1**	**1**	**0**	**2**	**1**	**0**	**0**	**0**
	• **Carbapenemase**	**0**	**0**	**0**	**0**	**0**	**0**	**0**	**0**	**0**	**0**	**0**	**0**	**0**	**0**	**0**
	• **Co-producers**	**0**	**0**	**0**	**0**	**0**	**0**	**0**	**0**	**0**	**0**	**0**	**0**	**0**	**0**	**0**
Unknowns	• **ESBL**	**1**	**6**	**0**	**0**	**1**	**1**	**1**	**1**	**0**	**0**	**1**	**1**	**0**	**0**	**0**
	• **Carbapenemase**	**1**	**8**	**0**	**0**	**1**	**1**	**1**	**1**	**0**	**0**	**1**	**1**	**0**	**1**	**1**
	• **Co-producers**	**0**	**0**	**0**	**0**	**0**	**0**	**0**	**0**	**0**	**0**	**0**	**0**	**0**	**0**	**0**

**a: N**_**T**_: total number of isolates in each phylogenetic group

b: **N:** number of pathogenicity islands

c: **ExPEC:** extra-intestinal pathogenic *E*. *coli*.

d: **DEC:** diarrheagenic *E*. *coli*

e: **PAIs:** pathogenicity islands.

For ExPEC PAIs in ESBL-producers, PAI IV_536_ was prevalent in groups B2 and D, while PAI II_J96_ was not detected at all. Regarding carbapenemase-producers, PAI III_536_, PAI IV_536_, and PAI II_CFT037_ were prevalent in group C. In contrast, PAI I_536_ and PAI II_J96_ were not detected. Concerning co-producers, PAI III_536_, PAI IV_536_, PAI I_CFT073_, PAI II_CFT037_, and PAI I_J96_ were distributed evenly in group D. In contrast, PAI I_536_, PAI II_536,_ and PAI II_J96_ were not detected.

## Discussion

*E*. *coli* causes many infectious diseases and treatment of these infections is a challenging situation due to antibiotic resistance. Nowadays, *E*. *coli* represents one of the most dangerous bacteria in the community, hospitals, and food. Therefore, the increase in resistance to antibiotics represents a risk and alarming factor that require fast handling of the situation.

Our study focused on antimicrobial resistance towards β-lactam antibiotics as they are the most used antibacterial agents. Our results of sensitivity patterns showed that the resistance was high in the environmental isolates against cefepime and meropenem compared to the clinical isolates. For amoxicillin-clavulanic acid, ceftazidime, and cefotaxime, the resistance was nearly the same in both categories. Besides, resistance to meropenem in the clinical and the environmental isolates (12.5% vs 24.2%, respectively) was higher than imipenem (6.9% vs 0%, respectively). In contrast, Abbas et al., 2019 reported that two *E*. *coli* (0.6%) and 15 *Klebsiella pneumoniae* (5%) out of 300 clinical isolates were carbapenem-resistant [26]. The increased resistance to cephalosporins may be attributed to the worldwide use of this class of antibiotics while carbapenems are still active in the treatment of serious infections [27].

Antibiotic resistance is mediated by several mechanisms. One of the primary mechanisms is the production of β-lactamase enzymes especially ESBLs and carbapenemases [28]. In this study, 53 isolates were ESBL-producers (62% clinical and 24% environmental isolates). The high percentage of ESBL-producers in the environmental isolates indicates the widespread of these enzymes from hospitals to society and the environment [9]. Although ESBL-producers were distributed among all clinical sources, the environmental isolates were restricted to only 3 sources (beef-burger, meat and milk).

Besides, modified Hodge test confirmed that the 18 resistant isolates to imipenem and/or meropenem were carbapenemase-producers. The phenotypic results obtained for ESBL and carbapenemase-producers were further confirmed by PCR which detects ESBL and carbapenemases encoding genes. Our study illustrated that *TEM*, *SHV* and *CTX-M15* were present in 100%, 88.7%, and 84.9% of ESBL-producers, respectively. The least detectable gene was multi-*TSO-O*. Similar results were reported previously [29]. For carbapenemase genes, *IMP*, *VIM*, and *OXA-48* genes were prevalent. All carbapenemase-encoding genes were distributed among the clinical and the environmental isolates except for *NDM* gene that was harbored only by the clinical isolates (P<0.0001). *IMP*, *VIM*, and *KPC* were significantly present in the environmental isolates (P<0.01). Therefore, food chains and human microbiota could be considered as new reservoirs for carbapenem resistance genes besides nosocomial bacteria. Liu et al. have reported similar results [30]. In contrast, Kalasseril et al. found that *VIM*, *IMP*, and GIM were absent among environmental *Enterobacteriaceae* isolates in India [31].

Seven patterns represented the ESBLs gene combinations **(Fig 3A).** *TEM* was the only gene that can be found either alone or in combinations. The combinations of ESBLs genes were high (96.2%) compared to the single ESBL gene pattern (3.8%). The clinical isolates were potential ESBL-producers as 51.1% of them carried all the tested ESBL genes. In environmental isolates, 37.5% of isolates carried the combination of (*TEM*+*SHV*+*CTX-M15*) indicating the importance of these isolates. Coproduction of carbapenemase genes was also found in clinical and environmental isolates in 10 different patterns **(Fig 3B)**. The majority of the environmental isolates harbored ≥3 genes and 50% of them carried four carbapenemase genes. These results illustrated the potential of food products as environmental sources of carbapenemase genes which necessitate strict food safety measures and public health regulations. Antibiotic resistance genes are considered a subtype of virulence factors that led to increased virulence and pathogenicity of these isolates [32, 33]. The highest co-production of *NDM* and *OXA-48* were limited to clinical isolates only and these isolates were resistant to all the tested β-lactams except one isolate that was only sensitive to imipenem.

All *E*. *coli* isolates were allocated to all pathotypes except enteroaggregative *E*. *coli* and diffusely adherent *E*. *coli* **(S5 and S6 Tables in S1 File)**. Canizalez-Roman et al. reported similar results [34]. EPEC and EHEC were prevalent among ESBL-producers (47.2%) and carbapenemase-producers (50%), respectively. Since 50% of the environmental isolates were EHEC, they were considered a gateway for dysentery and hemolytic uremic syndrome. Comparable results were reported earlier [35]. Besides, EIEC was the least common pathotype (3%) indicating its little role in diarrheal episodes in developing regions. This was consistent with the results of Hoseinzadeh et al. [36]. The ETEC pathotype among the environmental isolates (28.6%) was notable especially within beef burgers and meat, although ETEC was recognized as a waterborne pathogen. However, their detection in some food samples was recorded previously [37]. Indeed, food products readily available at public markets in Egypt were hypothesized to be involved in the transmission of the DEC foodborne illnesses [38].

Among the detected 24 serotypes within all isolates, O15:H2 and O103:H2 serotypes were predominant in ESBL- and carbapenemase-producers, respectively. The majority of them were related to EPEC and EHEC pathotypes, respectively. The serotypes O127:H6 and O128:H2 were restricted to ETEC. Although O127:H6 serotype is one of 12 EPEC serotypes recognized by the World Health Organization, it was one of the most common O serogroups reported in ETEC [39, 40]. All EIEC isolates lacked H flagellar antigen and belonged to O124 serotype.

PAIs were detected among 65 out of 66 *E*. *coli* isolates. For DEC PAIs, HPI island was the most abundant in both categories of our isolates indicating that HPI is a fitness island rather than a pathogenicity island [5, 19]. This was following Naderi et al. [19].

Five ExPEC PAIs were significantly detected in the clinical isolates of the ESBL and carbapenemase-producers **(Table 2).** Dobrindt et al. reported that these PAIs are more common in pathogenic *E*. *coli* than in commensal isolates [41]. Unlike the high prevalence of PAI IV536 marker in the majority of other studies stating that it is a chromosomal-stable island [42], our results demonstrated that PAI IICFT073 (98.5%) was the predominant marker in both ESBL- and carbapenemase-producers followed by PAI IV536 (92.4%) and PAI III536 (66.7%). A similar finding was reported previously [41]. The PAI I536 (2.9%) was the least common marker, distributed only in the clinical ESBL-producers where its acquisition and stabilization on the chromosome is very low [5].

It is of great interest to identify multiple PAIs (two to eight) in 97.14% of our isolates **(Table 3).** Isolates with a single PAI or without PAIs at all were rarely found (2.86%), while Naderi et al. showed that 24.19% of the isolates were with single or without PAIs [19]. Co-presence of DEC PAI markers were differentially distributed within the ESBL-producers (37.7%) and the carbapenemase-producers (38.9%) **(Fig 4).**

Interestingly, 100% of carbapenemase-producers carry either one ExPEC PAI (5.6%), or a combination (94.4%). Regarding the ESBL-producers, 94.3% carry either one or more Ex-PEC PAIs **(Fig 5).**

The phylogenetic analysis was conducted to create control and prevention programs and settle alternative treatments. Therefore, our study showed that D and B2 groups were predominant among the clinical ESBL-producers **(Fig 6A).** In contrast, the environmental ESBL-producers belonged to groups A, B1, and B2. Alizade et al. found that ESBL-positive isolates were mainly classified as groups A, D, and B2 [43]. In the carbapenemase-producers, the clinical isolates belonged to groups C (50%) and D (30%) while the environmental isolates were mainly groups B1, D, and A **(Fig 6B).** A previous study showed that carbapenemase positive isolates distributed in groups A, D, B2, and B1 in descending order [44]. Only one isolate was un-assigned to any phylogenetic group. This is consistent with the results reported by Clermont et al. [4]. In contrast, previous researchers reported higher percentages [15, 45]. These unassigned may be attributed to indistinct phylogroup or as a result of two varied phylogroup collections [4, 15].

The phylogenetic background of our *E*. *coli* pathotypes indicated that D is the major phylogroup followed by B2 among the ESBL-producers. The majority of the EPEC (33.3%) and the EHEC (26.3%) isolates are group D. Similar results were recorded by Ishii et al. [46]. Additionally, 50% of carbapenemase-producers are mainly group C of the EHEC pathotypes. DEC pathotypes have already been demonstrated to exhibit diverse phylogenetic origin [19, 47] but, to our knowledge, the link between phylogeny and serogroup has not yet been recognized.

In our results, the phylogenetic distribution showed to be strictly related to serogroups. Seven serotypes were limited to one specific phylogroup, regardless of their very few isolates. On the other hand, other serotypes are characterized by presenting a flexible phylogenetic distribution with a highly significant difference (P≤ 0.01).

Notably, PAIs markers were randomly distributed **(Table 4).** In ESBL-producers, the highest prevalence of DEC PAIs (HPI, Tia) and ExPEC PAIs (PAI III536, PAI IV536, PAI ICFT073and PAI IICFT073) belonged to group B2. Concerning carbapenemase-producers, the total prevalence of PAIs was in group C. Moreover, EspC PAI was found in groups D and U. In ESBL and carbapenemase coproducers, the distribution of PAIs were pervasive in group C. Moreover, isolates that harbored the maximal number of PAIs (8 markers), were in group B2 and unknown. This was reported in a previous study [5], which found more compatibility of PAI markers with B2 phylogroup.

In conclusion, the present study emphasizes the extremely high prevalence of ESBLs producers in the clinical and environmental *E*. *coli* isolates. Co-production of ESBLs encoding genes was greater in the clinical compared to the environmental isolates. The reverse scenario was observed for the co-production of carbapenemase encoding genes. Our study confirms the importance of phylogenetic groups D, B2, and C for antibiotic resistance in *E*. *coli*. Ultimately, our findings support a possible contribution of the environment to the local spread of *E*. *coli* pathogenicity in Egypt.

## Supporting information

S1 File (DOCX)
