# Correction: Genetic characterization of extended-spectrum β-Lactamase- and carbapenemase-producing *Escherichia coli* isolated from Egyptian hospitals and environments

**DOI:** 10.1371/journal.pone.0303719

**Published:** 2024-05-09

**Authors:** Soha El-Shaer, Shaymaa H. Abdel-Rhman, Rasha Barwa, Ramadan Hassan

Fig 1 was uploaded incorrectly. Please see the correct Fig 1 here.

**Fig 1:**
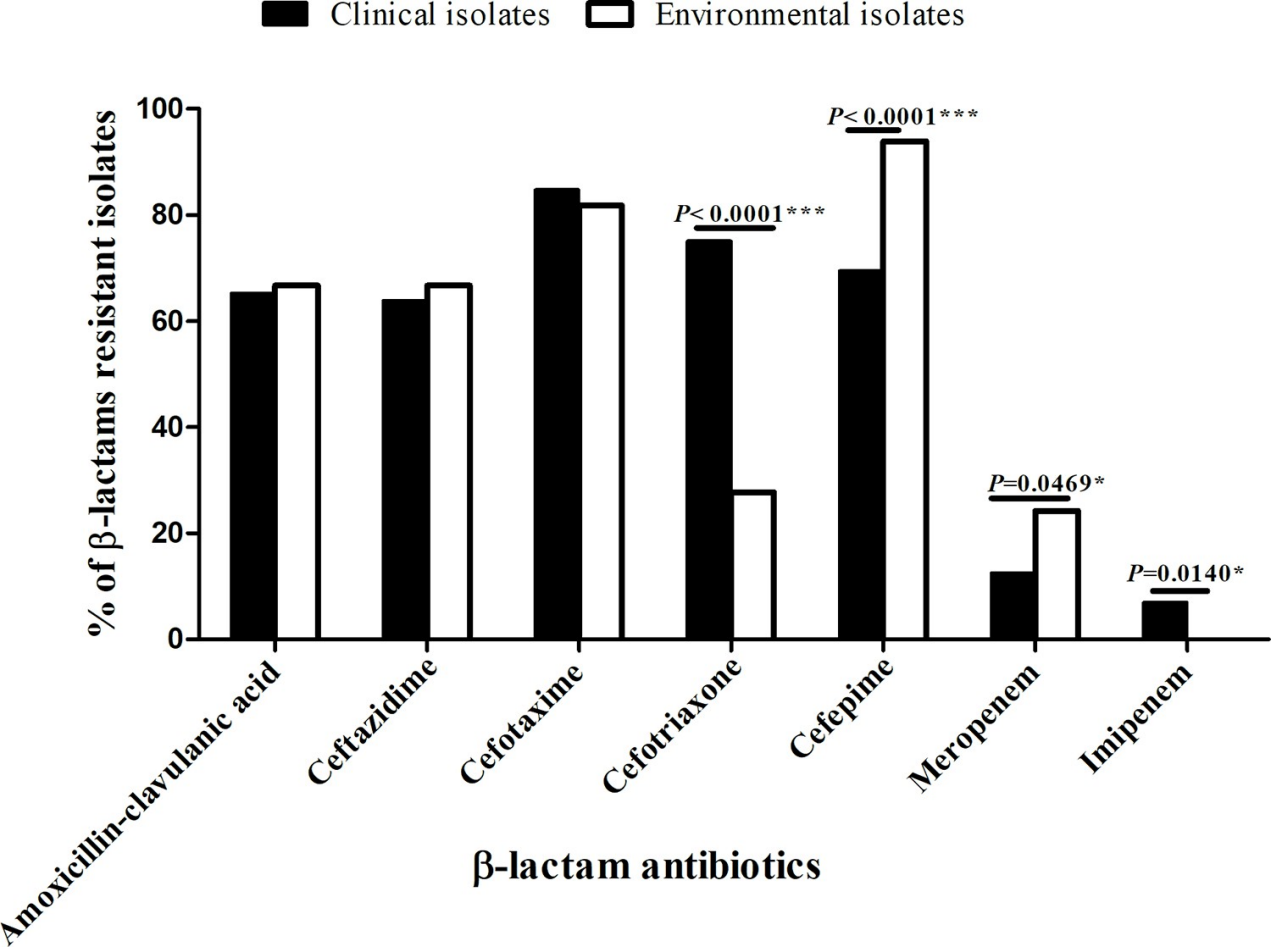
Comparison of β-lactams resistance level among the clinical and environmental *E*. *coli* isolates. (*significant, P< 0.05 and ***highly significant, P< 0.0001).
