# Supplementary material for: Genetic characterization of extended-spectrum β-Lactamase- and carbapenemase-producing Escherichia coli isolated from Egyptian hospitals and environments
Source: PLoS One. 2021 Jul 23;16(7):e0255219. doi: 10.1371/journal.pone.0255219 (PMC8301635; doi:10.1371/journal.pone.0255219)
Supplement: S1 File — (DOCX) [file pone.0255219.s001.docx]

**Supplementary material for on-line submission**

**S1 Table:** **The Polymerase chain reaction programs for amplification of ESBLs, carbapenemase resistant encoding genes and pathogenicity islands markers.**

| **Gene**  **type** | **Genes**  **name** | **Initial denaturation temp. (**^o^C**) /**  **time (mins)** | **Denaturation temp. (**^o^C**) / time** | | **Annealing temp. (**^o^C**) / time** | | **Extension temp. (**^o^C) **/ 1 mins** | | | **Final extension temp. (**^o^C**) / time (mins)** | | |
| --- | --- | --- | --- | --- | --- | --- | --- | --- | --- | --- | --- | --- |
|  |  | **1 cycle** | **40 cycles** | | | | | | | **1 cycle** | | |
| **Uniplex PCR for amplification of ESBLs and carbapenemase encoding genes** | | | | | | | | | | | | |
| **ESBLs** | *CTX-M-15* | 95/ 5 | | 95/ 30 secs | | 56 30 secs | | 72 | | 72/ 5 | | |
|  | *SHV* | 95/ 5 | | 95/ 30 secs | | 53/ 30 secs | | 72 | | 72/ 5 | | |
|  | *TEM* | 95/ 5 | | 95/ 30 secs | | 58/ 30 secs | | 72 | | 72/ 7 | | |
|  | *_TSO-O (OXA-1, -4, -30)_* | 95/ 5 | | 95/ 30 secs | | 60/ 30 secs | | 72 | | 72/ 5 | | |
| **Carbapenemase** | *NDM* | 94/ 5 | | 95/ 30 secs | | 52/ 30 secs | | 72 | | 72/ 5 | | |
|  | *OXA-48* | 95/ 5 | | 94/ 30 secs | | 57/ 30 secs | | 72 | | 72/ 5 | | |
|  | **Multiplex PCR for amplification of carbapenemase encoding genes** | | | | | | | | | | | |
|  | *IMP,*  *VIM* and  *KPC* | 94/5 | | 94/ 30 secs | | 55/ 30 secs | | 72 | | 72/ 7 | | |
| **Uniplex PCR for amplification of pathogenicity island markers** | | | | | | | | | | | | |
| **DEC PAIs markers** | *irp2* | 94/ 5 | 94/ 1 mins | | 61/ 1 mins | | 72 | | | 72/ 8 | | |
|  | *tia* | 94/ 5 | 94/ 1 mins | | 58/ 1 mins | | 72 | | | 72/ 8 | | |
|  | *efa/ lifA* | 94/ 5 | 94/ 1 mins | | 58/ 1 mins | | 72 | | | 72/ 8 | | |
|  | *pic* | 94/ 5 | 94/ 1 mins | | 57/ 1 mins | | 72 | | | 72/ 8 | | |
|  | *espC* | 94/ 5 | 94/ 1 mins | | 54/ 1 mins | | 72 | | | 72/ 8 | | |
| **Multiplex PCR for amplification of pathogenicity island markers** | | | | | | | | | | | |  |
| **ExPEC PAIs markers** | PAI I 536 and  PAI II 536 | 94/ 5 | | 94/ 1 mins | | 55/ 1 mins | | | 72 | | 72/ 10 |  |
|  | PAI III536 and  PAI IV536 | 95/ 5 | | 95/ 30 secs | | 55/ 30 secs | | | 72 | | 72/ 5 |  |
|  | PAI CFT073 I and  PAI CFT073 II | 95/ 5 | | 95/ 30 secs | | 56/ 30 secs | | | 72 | | 72/ 7 |  |
|  | PAI I J96 and  PAI II J96 | 94/ 5 | | 94/ 1 mins | | 53/ 1 mins | | | 72 | | 72/ 7 |  |

**S2 Table: Characterization of 72 clinical *E. coli* isolates based on collected data.**

| **Isolate code** | **Isolation center** | **Date of isolation** | **Isolate code** | **Isolation center** | **Date of isolation** | **Isolate code** | **Isolation center** | **Date of isolation** |
| --- | --- | --- | --- | --- | --- | --- | --- | --- |
| **Isolation source: urine (U)** | | | **Isolation source: rectum (R)** | | | **Isolation source: wound (W)** | | |
| **U1** | UNC | 19/10/2015 | **r1** | GEC | 19/10/2015 | **W1** | BCC | 31/10/2015 |
| **U2** | UNC | 19/10/2015 | **r2** | GEC | 19/10/2015 | **W2** | BCC | 31/10/2015 |
| **U3** | UNC | 19/10/2015 | **r3** | MEH | 26/11/2015 | **W3** | BCC | 31/10/2015 |
| **U4** | MIH | 26/10/2015 | **r4** | ICU | 13/12/2015 | **W4** | BCC | 11/11/2015 |
| **U5** | UNC | 26/10/2015 | **r5** | ICU | 13/12/2015 | **W5** | ICU | 13/12/2015 |
| **U6** | ICU | 26/10/2015 | **r6** | GEC | 19/12/2015 | **W6** | BCC | 13/12/2015 |
| **U7** | ICU | 26/10/2015 | **r7** | GEC | 19/12/2015 | **W7** | MIH | 26/12/2015 |
| **U8** | ICU | 2/1/2016 | **r8** | GEC | 19/12/2015 | **W8** | BCC | 13/1/2016 |
| **U9** | UNC | 13/1/2016 | **r9** | GEC | 19/12/2015 | **W9** | BCC | 25/1/2016 |
| **U10** | UNC | 25/1/2016 | **r10** | MUH | 25/1/2016 | **W10** | ICU | 25/1/2016 |
| **U11** | ICU | 25/1/2016 | **r11** | ICU | 3/2/2016 | **W11** | ICU | 23/2/2016 |
| **U12** | UNC | 29/2/2016 | **r12** | MUH | 6/2/2016 | **W12** | BCC | 13/3/2015 |
| **U13** | ICU | 5/3/2016 | **r13** | ICU | 15/2/2016 | **W13** | ICU | 13/3/2015 |
| **U14** | UNC | 5/3/2016 | **r14** | ICU | 15/2/2016 | **W14** | BCC | 13/3/2015 |
| **U15** | MUH | 5/3/2016 | **r15** | GEC | 29/3/2016 | **W15** | BCC | 25/1/2016 |
| **U16** | MUH | 8/3/2016 | **r16** | ICU | 30/3/2016 | **W16** | BCC | 25/1/2016 |
| **U17** | MEH | 8/3/2016 | **r17** | GEC | 13/4/2016 | **W17** | BCC | 23/2/2016 |
| **U18** | MUH | 16/3/2016 | **r18** | ICU | 13/4/2016 | **W18** | MIH | 13/3/2016 |
| **U19** | UNC | 30/3/2016 | **r19** | GEC | 27/4/2016 | **W19** | ICU | 13/3/2016 |
| **U20** | ICU | 12/4/2016 | **r20** | GEC | 19/5/2016 | **W20** | BCC | 13/3/2016 |
| **U21** | ICU | 13/4/2016 | **r21** | GEC | 25/5/2016 | **W21** | BCC | 28/4/2016 |
| **U22** | MEH | 13/4/2016 |  |  |  |  |  |  |
| **U23** | MIH | 17/4/2016 |  |  |  |  |  |  |
| **U24** | UNC | 17/4/2016 |  |  |  |  |  |  |
| **U25** | UNC | 17/4/2016 |  |  |  |  |  |  |
| **U26** | ICU | 19/4/2016 |  |  |  |  |  |  |
| **U27** | UNC | 19/4/2016 |  |  |  |  |  |  |
| **U28** | MIH | 28/4/2016 |  |  |  |  |  |  |
| **U29** | ICU | 9/5/2016 |  |  |  |  |  |  |
| **U30** | ICU | 25/5/2016 |  |  |  |  |  |  |

**U:** urine, **r:** rectal, **W:** wound, **UNC**: Urology and Nephrology Center, **MIH**: Mansoura International Hospital, **MEH:** Mansoura Emergency Hospital, **MUH**: Mansoura University Hospital, **GEC:** Gastroenterology Center, **BCC**: Burns and Cosmetics Center, **ICU:** Microbiology diagnostic Infection Control Unit.

**S3 Table: Characterization of 33 environmental *E. coli* isolates based on collected data.**

| **Isolate code** | **Isolation source** | **Isolation center** | **Date of isolation** | **Isolate code** | **Isolation source** | **Isolation center** | **Date of isolation** |
| --- | --- | --- | --- | --- | --- | --- | --- |
| **E1** | Pastrami meat | S1 | 10/10/2015 | **E18** | Milk | S3 | 2/3/2016 |
| **E2** | Luncheon meat | S2 | 19/10/2015 | **E19** | Ground beef | B3 | 2/3/2016 |
| **E3** | Healthy stool | Microbiology lab. FP, MU. | 31/10/2015 | **E20** | Ground beef | B1 | 2/3/2016 |
| **E4** | Sausage | S1 | 4/11/2015 | **E21** | Beef burger | S7 | 6/3/2016 |
| **E5** | Healthy stool | Microbiology lab. FP, MU. | 16/11/2015 | **E22** | Beef burger | S8 | 19/3/2016 |
| **E6** | Healthy stool | Microbiology lab. FP, MU. | 16/11/2015 | **E23** | Meat | B3 | 19/3/2016 |
| **E7** | Cheese | S3 | 16/11/2015 | **E24** | Ground beef | B2 | 19/3/2016 |
| **E8** | Healthy stool | Microbiology lab. FP, MU. | 30/11/2015 | **E25** | Beef burger | S8 | 31/3/2016 |
| **E9** | Cheese | S4 | 30/11/2015 | **E26** | Ground beef | B4 | 2/4/2016 |
| **E10** | Luncheon meat | S5 | 30/11/2015 | **E27** | Beef burger | B5 | 17/4/2016 |
| **E11** | Meat | B1 | 30/11/2015 | **E28** | Beef burger | S9 | 17/4/2016 |
| **E12** | Beef burger | S5 | 30/11/2015 | **E29** | Beef burger | B4 | 25/4/2016 |
| **E13** | Luncheon meat | S4 | 27/1/2016 | **E30** | Beef burger | B6 | 2/5/2016 |
| **E14** | Meat | B2 | 10/2/2016 | **E31** | Meat | B4 | 15/5/2016 |
| **E15** | Healthy stool | Microbiology lab. FP, MU. | 10/2/2016 | **E32** | Meat | B7 | 15/5/2016 |
| **E16** | Luncheon meat | S6 | 10/2/2016 | **E33** | Meat | B8 | 15/5/2016 |
| **E17** | Dill | S5 | 28/2/2016 |  | | | |

**E:** environmental, **S1 to S9:** different supermarkets, **B1 to B8:** different butcher shops,

**FP:** Faculty of Pharmacy, **MU:** Mansoura University.

**S4 Table: Antimicrobial susceptibility pattern of *E. coli* isolated from clinical and environmental sources.**

| **Isolate code** | **Amoxicillin-clavulanic acid** | **3rd generation cephalosporins** | | | **4th generation cephalosporins** | **Carbapenems** | |
| --- | --- | --- | --- | --- | --- | --- | --- |
|  | **AMC** | **CAZ** | **CTX** | **CRO** | **FEP** | **MEM** | **IPM** |
| **U1** | **R** | **R** | **R** | **R** | **R** | **S** | **S** |
| **U2** | **R** | **R** | **R** | **R** | **R** | **S** | **S** |
| **U3** | **S** | **S** | **S** | **S** | **S** | **S** | **S** |
| **U4** | **R** | **R** | **R** | **R** | **R** | **S** | **S** |
| **U5** | **R** | **S** | **S** | **S** | **S** | **S** | **S** |
| **U6** | **I** | **R** | **R** | **R** | **R** | **S** | **S** |
| **U7** | **I** | **R** | **R** | **R** | **R** | **S** | **S** |
| **U8** | **R** | **R** | **R** | **R** | **R** | **S** | **S** |
| **U9** | **R** | **R** | **R** | **R** | **R** | **S** | **S** |
| **U10** | **R** | **R** | **R** | **R** | **R** | **S** | **S** |
| **U11** | **S** | **R** | **R** | **R** | **R** | **S** | **S** |
| **U12** | **S** | **R** | **R** | **R** | **R** | **S** | **I** |
| **U13** | **R** | **R** | **R** | **R** | **R** | **S** | **S** |
| **U14** | **R** | **R** | **R** | **R** | **R** | **S** | **S** |
| **U15** | **R** | **I** | **I** | **R** | **R** | **S** | **S** |
| **U16** | **S** | **S** | **S** | **S** | **S** | **S** | **S** |
| **U17** | **R** | **R** | **R** | **R** | **R** | **S** | **S** |
| **U18** | **S** | **I** | **R** | **R** | **R** | **S** | **S** |
| **U19** | **I** | **S** | **S** | **S** | **S** | **S** | **S** |
| **U20** | **R** | **R** | **R** | **R** | **SDD** | **S** | **S** |
| **U21** | **R** | **R** | **R** | **R** | **R** | **S** | **S** |
| **U22** | **S** | **S** | **S** | **S** | **S** | **S** | **S** |
| **U23** | **R** | **I** | **R** | **R** | **R** | **I** | **R** |
| **U24** | **I** | **R** | **R** | **R** | **R** | **S** | **S** |
| **U25** | **I** | **R** | **R** | **R** | **R** | **S** | **S** |
| **U26** | **I** | **R** | **R** | **R** | **R** | **S** | **S** |
| **U27** | **R** | **R** | **R** | **R** | **R** | **S** | **S** |
| **U28** | **R** | **R** | **R** | **R** | **R** | **S** | **S** |
| **U29** | **I** | **S** | **S** | **S** | **SDD** | **S** | **S** |
| **U30** | **S** | **S** | **S** | **S** | **SDD** | **S** | **S** |
| **r1** | **R** | **R** | **R** | **R** | **R** | **S** | **S** |
| **r2** | **I** | **I** | **R** | **R** | **R** | **S** | **S** |
| **r3** | **R** | **R** | **R** | **R** | **R** | **S** | **S** |
| **r4** | **R** | **R** | **R** | **R** | **R** | **R** | **I** |
| **r5** | **R** | **R** | **R** | **R** | **R** | **S** | **S** |
| **r6** | **S** | **S** | **S** | **S** | **S** | **S** | **S** |
| **r7** | **R** | **R** | **R** | **R** | **R** | **R** | **R** |
| **r8** | **R** | **R** | **R** | **R** | **R** | **S** | **S** |
| **r9** | **R** | **R** | **R** | **R** | **SDD** | **S** | **S** |
| **r10** | **R** | **R** | **R** | **R** | **R** | **S** | **S** |
| **r11** | **R** | **R** | **R** | **R** | **R** | **R** | **R** |
| **r12** | **R** | **R** | **R** | **R** | **R** | **R** | **R** |
| **r13** | **I** | **I** | **I** | **R** | **SDD** | **S** | **S** |
| **r14** | **R** | **R** | **R** | **R** | **SDD** | **S** | **S** |
| **r15** | **S** | **S** | **S** | **S** | **S** | **S** | **S** |
| **r16** | **R** | **R** | **R** | **R** | **R** | **S** | **S** |
| **r17** | **R** | **R** | **R** | **R** | **R** | **S** | **S** |
| **r18** | **R** | **R** | **R** | **R** | **R** | **R** | **S** |
| **r19** | **R** | **S** | **R** | **S** | **SDD** | **I** | **S** |
| **r20** | **R** | **R** | **R** | **R** | **R** | **R** | **S** |
| **R21** | **S** | **S** | **R** | **S** | **SDD** | **S** | **S** |
| **W1** | **R** | **R** | **R** | **R** | **R** | **I** | **S** |
| **W2** | **I** | **R** | **R** | **R** | **R** | **S** | **S** |
| **W3** | **R** | **R** | **R** | **R** | **R** | **S** | **S** |
| **W4** | **I** | **R** | **R** | **R** | **R** | **S** | **S** |
| **W5** | **R** | **R** | **R** | **R** | **R** | **I** | **S** |
| **W6** | **R** | **S** | **R** | **S** | **SDD** | **S** | **S** |
| **W7** | **R** | **R** | **R** | **R** | **R** | **R** | **R** |
| **W8** | **R** | **S** | **R** | **S** | **SDD** | **I** | **S** |
| **W9** | **R** | **R** | **R** | **R** | **R** | **R** | **S** |
| **W10** | **R** | **R** | **R** | **R** | **R** | **R** | **S** |
| **W11** | **I** | **I** | **R** | **R** | **R** | **I** | **S** |
| **W12** | **S** | **S** | **R** | **S** | **SDD** | **S** | **S** |
| **W13** | **R** | **S** | **R** | **S** | **SDD** | **S** | **S** |
| **W14** | **R** | **R** | **R** | **R** | **R** | **S** | **S** |
| **W15** | **R** | **I** | **R** | **R** | **R** | **I** | **S** |
| **W16** | **R** | **S** | **R** | **R** | **R** | **I** | **S** |
| **W17** | **I** | **R** | **R** | **R** | **R** | **S** | **S** |
| **W18** | **R** | **S** | **R** | **S** | **S** | **S** | **S** |
| **W19** | **R** | **S** | **R** | **S** | **S** | **S** | **S** |
| **W20** | **R** | **R** | **R** | **R** | **R** | **I** | **S** |
| **W21** | **S** | **S** | **R** | **S** | **SDD** | **S** | **S** |
| **E1** | **R** | **I** | **R** | **I** | **R** | **I** | **S** |
| **E2** | **R** | **R** | **R** | **R** | **R** | **R** | **S** |
| **E3** | **R** | **I** | **R** | **R** | **R** | **I** | **S** |
| **E4** | **R** | **I** | **R** | **S** | **R** | **I** | **S** |
| **E5** | **R** | **I** | **R** | **I** | **R** | **R** | **S** |
| **E6** | **R** | **R** | **R** | **I** | **R** | **R** | **S** |
| **E7** | **R** | **R** | **R** | **I** | **R** | **R** | **S** |
| **E8** | **R** | **R** | **R** | **I** | **R** | **I** | **S** |
| **E9** | **R** | **R** | **R** | **I** | **R** | **R** | **S** |
| **E10** | **R** | **R** | **R** | **I** | **R** | **S** | **S** |
| **E11** | **R** | **S** | **R** | **I** | **R** | **I** | **S** |
| **E12** | **R** | **R** | **R** | **I** | **R** | **S** | **S** |
| **E13** | **R** | **R** | **R** | **I** | **R** | **I** | **S** |
| **E14** | **R** | **R** | **R** | **S** | **R** | **I** | **S** |
| **E15** | **R** | **R** | **R** | **I** | **R** | **R** | **S** |
| **E16** | **R** | **S** | **R** | **S** | **R** | **I** | **S** |
| **E17** | **R** | **I** | **R** | **I** | **R** | **I** | **S** |
| **E18** | **R** | **S** | **R** | **I** | **R** | **S** | **S** |
| **E19** | **R** | **S** | **R** | **S** | **R** | **I** | **S** |
| **E20** | **I** | **S** | **R** | **I** | **R** | **I** | **S** |
| **E21** | **S** | **R** | **I** | **S** | **R** | **R** | **S** |
| **E22** | **I** | **R** | **R** | **R** | **R** | **I** | **S** |
| **E23** | **S** | **R** | **I** | **S** | **R** | **I** | **S** |
| **E24** | **S** | **R** | **I** | **S** | **R** | **I** | **S** |
| **E25** | **R** | **R** | **R** | **R** | **R** | **I** | **S** |
| **E26** | **I** | **R** | **I** | **S** | **R** | **I** | **S** |
| **E27** | **S** | **R** | **R** | **R** | **R** | **I** | **S** |
| **E28** | **S** | **R** | **I** | **S** | **R** | **S** | **S** |
| **E29** | **I** | **R** | **R** | **R** | **R** | **S** | **S** |
| **E30** | **I** | **R** | **R** | **R** | **R** | **R** | **S** |
| **E31** | **I** | **S** | **S** | **S** | **SDD** | **S** | **S** |
| **E32** | **R** | **R** | **R** | **R** | **SDD** | **S** | **S** |
| **E33** | **R** | **R** | **R** | **R** | **R** | **S** | **S** |

**U:** urine, **r:** rectal, **W**: wound, **E:** environmental  **R:** resistant, **I:** intermediate, **SDD:** susceptible-dose dependent, **S:** sensitive**AMC:** amoxicillin-clavulanic, **CAZ:** ceftazidime, **CTX:** cefotaxime, **CRO:** cefotriaxone, **FEP:** cefepime, **MEM:** meropenem, **IPM:** imipenem.

**S5 Table: Serotyping and distribution of extended-spectrum β-lactamase encoding genes among fifty-three *E. coli* isolates**

| **Isolate code** | ***bla***  ***_CTX-M-15_*** | ***bla _SHV_*** | ***bla _TEM_*** | ***bla _TSO-O (OXA-1, -4, -30)_*** | Serotype | pathotype | **Isolate code** | ***bla _CTX-M-15_*** | ***bla _SHV_*** | ***bla _TEM_*** | ***bla _TSO-O (OXA-1, -4, -30)_*** | Serotype | pathotype |
| --- | --- | --- | --- | --- | --- | --- | --- | --- | --- | --- | --- | --- | --- |
| **Clinical isolates** | | | | | | | | | | | | | |
| **U1** | **+** | **+** | **+** | **-** | O8 : H21 | EPEC | **r13** | **+** | **+** | **+** | **-** | O119 : H6 | EPEC |
| **U2** | **+** | **+** | **+** | **+** | O44 : H18 | EHEC | **r16** | **+** | **+** | **+** | **-** | O91 : H21 | EHEC |
| **U3** | **+** | **+** | **+** | **+** | O15 : H2 | EPEC | **r17** | **-** | **-** | **+** | **+** | O15 : H2 | EPEC |
| **U4** | **+** | **+** | **+** | **+** | O15 : H2 | EPEC | **r19** | **-** | **+** | **+** | **-** | O127 : H6 | ETEC |
| **U6** | **+** | **+** | **+** | **+** | O75 | ETEC | ***r20** | **+** | **+** | **+** | **+** | O91 : H21 | EHEC |
| **U7** | **+** | **+** | **+** | **+** | O7 : H2 | EPEC | **W1** | **+** | **+** | **+** | **+** | O127 : H6 | ETEC |
| **U8** | **-** | **+** | **+** | **-** | O2 : H6 | EPEC | **W2** | **-** | **+** | **+** | **+** | O55 : H7 | EPEC |
| **U9** | **+** | **+** | **+** | **+** | O83 | EPEC | **W4** | **+** | **+** | **+** | **+** | O127 : H6 | ETEC |
| **U10** | **+** | **+** | **+** | **+** | O15 : H2 | EHEC | **W5** | **+** | **+** | **+** | **+** | O55 : H7 | EPEC |
| **U11** | **+** | **+** | **+** | **+** | O8 : H21 | EPEC | ***W9** | **+** | **+** | **+** | **+** | O78 | EPEC |
| **U12** | **+** | **+** | **+** | **+** | O1 : H7 | EHEC | ***W10** | **+** | **+** | **+** | **-** | O124 | EIEC |
| **U13** | **+** | **+** | **+** | **+** | O2 : H6 | EPEC | **W11** | **+** | **+** | **+** | **-** | O44 : H18 | EHEC |
| **U14** | **+** | **+** | **+** | **+** | O15 : H2 | EHEC | **W14** | **+** | **+** | **+** | **+** | O127 : H6 | ETEC |
| **U17** | **+** | **+** | **+** | **+** | O75 | EHEC | **W15** | **+** | **+** | **+** | **+** | O114 : H4 | EPEC |
| **U18** | **+** | **+** | **+** | **+** | O2 : H6 | EPEC | **W16** | **+** | **+** | **+** | **-** | O7 : H2 | EPEC |
| **U24** | **+** | **+** | **+** | **-** | O7 : H2 | EPEC | **W17** | **-** | **-** | **+** | **-** | O55 : H7 | EHEC |
| **U25** | **+** | **+** | **+** | **-** | O8 : H21 | EPEC | **W19** | **+** | **+** | **+** | **-** | O171 : H2 | EPEC |
| **U26** | **+** | **+** | **+** | **-** | O2 : H6 | EHEC | **W21** | **-** | **+** | **+** | **-** | O55 : H7 | EHEC |
| **U27** | **+** | **+** | **+** | **+** | O83 | EPEC | **Environmental isolates** | | | | | | |
| **U28** | **+** | **+** | **+** | **+** | O26 : H11 | EHEC | ***E21** | **+** | **+** | **+** | **-** | *O126 : H21* | *ETEC* |
| **r1** | **+** | **+** | **+** | **+** | O91 : H21 | EHEC | **E22** | **+** | **-** | **+** | **-** | *O91 : H21* | *EHEC* |
| **r2** | **+** | **+** | **+** | **+** | O113 : H4 | EPEC | **E23** | **+** | **+** | **+** | **-** | *O128 : H2* | *ETEC* |
| **r3** | **+** | **+** | **+** | **+** | O26 : H11 | EHEC | **E25** | **+** | **+** | **+** | **+** | *O26 : H11* | *EHEC* |
| **R5** | **+** | **+** | **+** | **+** | O128 : H2 | ETEC | **E27** | **+** | **-** | **+** | **-** | *O44 : H18* | *EPEC* |
| **R8** | **+** | **+** | **+** | **+** | O124 | EIEC | **E29** | **-** | **-** | **+** | **-** | *O113 : H4* | *EPEC* |
| **R9** | **-** | **+** | **+** | **+** | O111 : H2 | EHEC | ***E30** | **+** | **-** | **+** | **+** | *O126 : H21* | *ETEC* |
| **R10** | **+** | **+** | **+** | **+** | O8 : H21 | EPEC | **E33** | **+** | **+** | **+** | **-** | *O91 : H21* | *EHEC* |

* Isolates co-produce ESBLs and carbapenamases encoding genes

**S6 Table: Serotyping and distribution of carbapenemase encoding genes among eighteen *E. coli* isolates**

| **Isolate code** | **Multiplex PCR** | | | **Simplex PCR** | | Serotype | Seropathotype |
| --- | --- | --- | --- | --- | --- | --- | --- |
|  | ***bla _IMP_*** | ***bla _VIM_*** | ***bla _KPC_*** | ***bla _NDM-1_*** | ***bla _OXA-48_*** |  |  |
| **Clinical isolates** | | | | | | | |
| **U23** | **+** | **+** | **-** | **-** | **+** | O15 : H2 | EPEC |
| **r4** | **+** | **+** | **-** | **-** | **+** | O127 : H6 | ETEC |
| **r7** | **-** | **-** | **-** | **+** | **+** | O103 : H2 | EHEC |
| **r11** | **+** | **+** | **+** | **+** | **+** | O55 : H7 | EHEC |
| **r12** | **-** | **+** | **+** | **+** | **+** | O26 : H11 | EHEC |
| **r18** | **+** | **+** | **-** | **-** | **+** | O103 : H2 | EHEC |
| ***r20** | **+** | **-** | **-** | **-** | **+** | O91 : H21 | EHEC |
| **W7** | **+** | **+** | **-** | **+** | **+** | O124 | EIEC |
| ***W9** | **+** | **+** | **-** | **+** | **+** | O78 | EPEC |
| ***W10** | **+** | **-** | **-** | **+** | **-** | O124 | EIEC |
| **Environmental isolates** | | | | | | | |
| **E2** | **+** | **+** | **-** | **-** | **+** | O121 : H7 | EHEC |
| **E5** | **+** | **+** | **+** | **-** | **+** | O111 : H2 | EHEC |
| **E6** | **+** | **+** | **+** | **-** | **+** | O113 : H4 | EPEC |
| **E7** | **+** | **+** | **+** | **-** | **+** | O78 | EPEC |
| **E9** | **+** | **+** | **-** | **-** | **+** | O55 : H7 | EHEC |
| **E15** | **+** | **-** | **+** | **-** | **+** | O128 : H2 | ETEC |
| ***E21** | **+** | **+** | **+** | **-** | **-** | O103 : H2 | EHEC |
| ***E30** | **+** | **+** | **+** | **-** | **+** | O44 : H18 | EPEC |

* Isolates co-produce ESBLs and carbapenamases encoding genes
